# An In Situ XAS Study of the Cobalt Rhenium Catalyst for Ammonia Synthesis

**DOI:** 10.1007/s11244-018-0892-7

**Published:** 2018-02-12

**Authors:** Karina Mathisen, Karsten Granlund Kirste, Justin S. J. Hargreaves, Said Laassiri, Kate McAulay, Andrew R. McFarlane, Nicholas A. Spencer

**Affiliations:** 1 Department of Chemistry, Norwegian University of Science and Technology (NTNU), Høgskoleringen 5, 7491 Trondheim, Norway; 2 WestCHEM, School of Chemistry, University of Glasgow, Joseph Black Building, Glasgow, G12 8QQ UK

**Keywords:** Ammonia, Cobalt, Rhenium, In situ XAS

## Abstract

A cobalt rhenium catalyst active for ammonia synthesis at 400 °C and ambient pressure was studied using in situ XAS to elucidate the reducibility and local environment of the two metals during reaction conditions. The ammonia reactivity is greatly affected by the gas mixture used in the pre-treatment step. Following H_2_/Ar pre-treatment, a subsequent 20 min induction period is also observed before ammonia production occurs whereas ammonia production commences immediately following comparable H_2_/N_2_ pre-treatment. In situ XAS at the Co K-edge and Re L_III_-edge show that cobalt initiates reduction, undergoing reduction between 225 and 300 °C, whereas reduction of rhenium starts at 300 °C. The reduction of rhenium is near complete below 400 °C, as also confirmed by H_2_-TPR measurements. A synergistic co-metal effect is observed for the cobalt rhenium system, as complete reduction of both cobalt and rhenium independently requires higher temperatures. The phases present in the cobalt rhenium catalyst during ammonia production following both pre-treatments are largely bimetallic Co–Re phases, and also monometallic Co and Re phases. The presence of nitrogen during the reduction step strongly promotes mixing of the two metals, and the bimetallic Co–Re phase is believed to be a pre-requisite for activity.

**Electronic supplementary material:**

The online version of this article (10.1007/s11244-018-0892-7) contains supplementary material, which is available to authorized users.

## Introduction

Since the development of the Haber Bosch Process at the beginning of the twentieth Century, considerable effort has been made to reduce the severity of the process by developing new efficient catalytic materials operational under milder reaction conditions [1–5]. The current industrial catalyst which is based on promoted iron is operated at high pressure (ca. 100–300 atm.) and moderate temperatures (ca. 400 °C) to ensure acceptable industrial ammonia production rates [6]. Amongst the materials developed as alternatives to the iron based system, Re based catalysts display comparatively high catalytic activity [7]. The catalytic activity of such Re based catalysts was found only to be stabilized when cobalt was added as a promoter to the bulk Re (atomic ratio of 4:1 Re:Co), whereas rapid deactivation over time was observed in the case of the non-promoted system [8]. The stability of the cobalt rhenium phase was correlated in the literature to the formation of a stable active rhenium nitride phase upon the ammonolysis step employed in its preparation. Subsequently, more recent work from McAulay et al. [9] has demonstrated that highly active cobalt rhenium catalysts can be prepared without an ammonolysis stage. Interestingly, a pronounced influence of pre-treatment gas mixture was reported in the catalytic activity of this material with 75% H_2_ in N_2_ leading to more active catalyst than when applying 75% H_2_ in Ar. Furthermore, an induction period of 20 min was observed before the catalytic activity developed in the case of the H_2_ in Ar pre-treated catalyst [9]. Although, post-reaction analysis did not reveal any relevant structural changes in cobalt rhenium upon the different pre-treatments, subtle changes, not easily detected by classical characterization techniques, might be responsible for these differences.

In situ X-ray absorption spectroscopy (XAS) has been reported in the literature as an efficient tool for studying the rhenium cobalt catalyst used in the Fischer–Tropsch Process [10–15]. Since XAS is an element specific probe, information related to the local environment of each constituent, Co or Re, can be obtained in an independent manner [16]. The Near Edge (XANES) region is highly sensitive to local environment and oxidation state, meaning fingerprinting with carefully selected reference compounds can reveal initial information about the state of the elements involved. From the extended fine structure region (EXAFS) structural information, inaccessible by standard characterization techniques, can be obtained about the local environment (up to a distance of 4 Å) surrounding the absorbing atom. For instance rhenium promoted Co/Al_2_O_3_ was studied via in situ EXAFS at the Re L_III_ (10535 eV) and Co K (71676 eV) edges [17]. Analysis of both edges indicated that the Re was atomically distributed inside bulk Co particles. At the Re L_III_ edge an increased Re–Co interatomic distance was found (in comparison to a model based on DTF calculations by Bakken et al. [18]) and this was attributed to a distortion of the local Co environment for the Co particles which were in direct contact with Re particles. This distortion was only evident at the Re L_III_ edge and was not found at the Co K-edge or by XRD analysis [19]. Bimetallic Re–Co interactions during the reduction of Co–Re/Al_2_O_3_ (2 wt% Co and 4.6 wt% Re) were also studied by Rønning et al. [15]. Insight into the reduction behaviour of Re and Co was obtained using in situ EXAFS analysis. The reduction of rhenium was found to occur subsequently to the reduction of cobalt. The reduction process resulted in the formation of bimetallic Re–Co species with Re–Co distances found to be approximately 2.53 Å.

In this paper, the structural evolution of the cobalt rhenium system resulting from the different pre-treatments and during the induction period has been examined by in situ XAS using conditions mimicking ammonia synthesis conditions. Figures denoted by S within the text can be found in the Supplementary Information.

## Experimental

### Cobalt Rhenium Synthesis and Preparation of References

Cobalt rhenium was prepared by incipient wetness impregnation of NH_4_ReO_4_ (Sigma Aldrich, 99%) with Co(NO_3_)_2_·6H_2_O (Sigma Aldrich, 98%) to yield a synthesis Co/Re ratio of 1/4, as previously described [9]. The sample was dried overnight and calcined in air at 700 °C for 3 h. Inductively Coupled Plasma-Mass Spectroscopy (ICP-MS) elemental analysis was conducted using a High Resolution Inductively Coupled Plasma MS Element 2 from Thermo Scientific. The samples (10–20 mg) were decomposed by adding concentrated nitric acid (HNO_3_, 1.5 ml) and hydrofluoric acid (HF, 40 wt%, 0.6 g). The resultant solution was diluted with deionized water until a total weight of 216.6 g was reached.

Nitrided cobalt (CoN_x_) was prepared by reaction of ammonia with Co_3_O_4_ at 700 °C for 2 h. The temperature was increased from room temperature to 300 °C over 30 min, after which it was increased to 450 °C at a rate of 0.7 °C min^− 1^ and then up to 700 °C at a rate of 1.67 °C min^− 1^. Nitrided rhenium (ReN_x_) was prepared by ammonolysis of NH_4_ReO_4_ (Sigma Aldrich, 99.5%) at 350 °C for 2 h. A temperature ramp rate of 5 °C min^− 1^ was applied. Nitrided cobalt rhenium (CoRe_y_N_x_) was prepared by ammonolysis of a cobalt rhenium oxide precursor, prepared as described above, at 700 °C for 3 h. A temperature ramp rate of 5 °C min^− 1^ was applied.

### Denitridation

The reactivity of cobalt rhenium was probed, using 0.3 g of catalyst, under an atmosphere of 75% H_2_ in Ar (BOC, 99.98%) at a total gas feed of 60 ml min^− 1^. Prior to reaction, the cobalt rhenium material was activated under an atmosphere of 75% H_2_ in N_2_ (BOC, 99.98%) mixture at a total gas feed of 60 ml min^− 1^ at 600 °C for 2 h. Ammonia production applying a 60 ml min^− 1^ flow of 75% H_2_ in Ar at 400 °C was determined by measurement of the decrease in conductivity of a 200 ml 0.0018 M H_2_SO_4_ solution through which the reactor effluent stream was flowed.

### Temperature Programmed Reduction

The reducibility of cobalt rhenium samples was studied by temperature programmed reduction (TPR). 40 mg of sample was pre-treated under 5.0 vol% O_2_ in He using a flow rate of 50 ml min^− 1^ at 600 °C for 2 h, and then cooled down to ambient temperature. Thereafter, the TPR experiment was performed under a mixture of gases composed of 5.0 vol% H_2_ in He using a total gas feed of 50 ml min^− 1^ with a temperature ramp rate of 5 °C min^− 1^.

### XAS Data Collection

Cobalt K-edge and rhenium L_III_-edge XAS data was collected in transmission mode at the Swiss-Norwegian Beamlines (SNBL, BM1B) at the European Synchrotron Radiation Facility (ESRF). The data was collected in the 16-bunch filling mode, providing a maximum current of 90 mA. A bending magnet collects the white beam from the storage ring to the beamline. BM01B is equipped with a Si(111) double crystal monochromator for EXAFS data collection. The incident and transmitted intensities (I_0_ and I_t_ + I_2_) were detected with ion chambers filled with, I_0_ (17 cm) 50% N_2_ + 50% He, and I_t_ and I_2_ (30 cm) with 85% N_2_ + 15% Ar at the cobalt edge. Post-ammonolysis samples (CoN_x,_ ReN_x_ and CoReN_x_), cobalt references (CoO, Co_3_O_4_) and rhenium references (ReO_3_, KReO_4_ and NH_4_ReO_4_) placed in aluminium sample holders were also collected ex situ and were mixed with boron nitride for optimum absorption. The cobalt XAS data were measured in continuous step scan from 7600 to 8300 eV with a step size of 0.5 eV and counting time 300 ms. The rhenium L_III_-edge data was collected in transmission mode, using ion chambers fillings 100% N_2_ (I_0,_ 17 cm), 50% N_2_ + 50% Ar (I_t_ and I_2_, 30 cm). Step scans were collected between 10,350 and 11,800 eV, with a step size of 0.5 eV and counting time 200 ms.

For all in situ measurements, great care was taken to ensure that similar conditions were applied for both edges and hence sample weight, cell thickness and gas flow were kept constant. The cobalt rhenium catalyst was mixed with boron nitride, pressed into wafers and sieved. Sieved samples (above 375 μm) were then placed inside 0.9 mm quartz capillaries with quartz wool on either side. The capillary was heated by a blower placed directly under the sample, and the exhaust was continuously sampled using a Tristar Mass spectrometer.

Samples were studied using two protocols with pre-treatments either in 75% H_2_ in Ar or 75% H_2_ in N_2_ at 600 °C for 2 h using a 10 °C min^− 1^ temperature ramp rate with a total gas flowrate of 10 ml min^− 1^. EXAFS step scans were collected continuously following the protocol shown in Figure S.1 with XRD patterns being collected at the end points. After the pre-treatment, samples were cooled to 400 °C and the reaction gas was switched to 75% H_2_ in N_2_. EXAFS step scans were collected continuously. A setup with pneumatic switching valves and a secondary by-pass line was employed to prevent gas build-up during switching between gases.

### XAS Data Refinements

The XAS data was binned (edge region − 30 to 50 eV; pre-edge grid 10 eV; XANES grid 0.5 eV; EXAFS grid 0.05 Å^−1^) and background subtracted, and the EXAFS part of the spectrum extracted to yield the χ_i_^exp^(k) using Athena software from the IFFEFITT package. The XANES spectra were normalised from 30 to 150 eV above the edge, while the EXAFS spectra were normalised from 150 eV to the end point [20]. The data were carefully deglitched and truncated at the end of the EXAFS spectra when needed. The smooth background μ_0_(E) was checked and corrected to achieve the maximum overlap with total absorption μ(E). Athena uses the Autobk algorithm to determine the background and normalise μ(E) data. Edge step normalisation was used, meaning that the difference between μ(E) and μ_0_(E) was divided by an estimation of μ_0_(E_0_). A line was regressed to the data in the region below the edge and subtracted from the data. A quadratic polynomial was then regressed to the data above the edge and extrapolated back to E_0_ [20]. For cobalt the threshold energy (E_0_) was set to be at the mid-point (0.5) of the normalised absorption edge step ensuring it was chosen after any pre-edge or shoulder features. For rhenium samples E_0_ was determined to be the first inflection point in the first derivative spectra, as there are no pre-edges or shoulder features. All XANES spectra are energy corrected against the corresponding reference foil (Co = 7709 eV, Re = 10,535 eV).

XANES is invaluable for distinguishing between valence states of transition metals and is highly sensitive towards changes in the electronic states and or the local environment. At every stage of the process, attempts were made to fit the experimental spectra to reference compounds, including corresponding foils, using the linear combination feature in the Athena package [20]. The normalised data were fitted from − 20 eV below the edge and 60 eV above the edge, allowing also the E_0_ value to be fitted, due to the ambiguity in selecting this value resulting from pre-edge features. All fits with unreasonable ΔE_0_ values (± 2.5 eV) were excluded. The maximum number of standards was set to three and the most relevant were chosen on the basis of the statistical goodness of fit.

EXAFS least-squares refinements were carried out using DL-EXCURV [21], which conducts the curve fitting of the theoretical *χ*^th^(*k*) to the experimental *χ*^exp^(*k*) using the curved wave theory. The fit parameter reported for each refinement procedure is given by the statistical R-factor, defined as:

$$R=\sum\limits_{i}^{N} {\left[ {\frac{1}{{{\sigma _i}}}\left( {|\chi _{i}^{{\exp }}\left( k \right) - ~\chi _{i}^{{th}}\left( k \right)|} \right)} \right]} ~\; \times 100\%$$

Ab initio phase shifts for both edges were also calculated within DL-EXCURV and verified using reference compounds. The amplitude reduction factor (AFAC) was extracted from the following reference compounds: Co_3_O_4_ and NH_4_ReO_4_ for the oxidized cobalt rhenium phase, while Co-foil and Re-foil were used for the reduced phase. The least-squares refinements were carried out in typical wave number k range 2–8.5 Å^−1^ for cobalt and k range 3.5–9.5 Å^−1^ for rhenium using a k^3^ weighting scheme.

## Results

### Cobalt Rhenium Starting Material

The original catalyst was labelled according to the original synthesis molar ratio between the cobalt and rhenium (CoRe_4_). However, in order to elucidate the real composition of the material ICP-MS was performed and such analysis revealed the actual molar composition to be CoRe_1.6_. The loss of rhenium from the sample may be attributed to volatilization of rhenium during the calcination stage at 700 °C in air prior to reaction. From here on in within this manuscript the cobalt rhenium catalyst will be labelled as CoRe_1.6_.

**Fig. 1:**
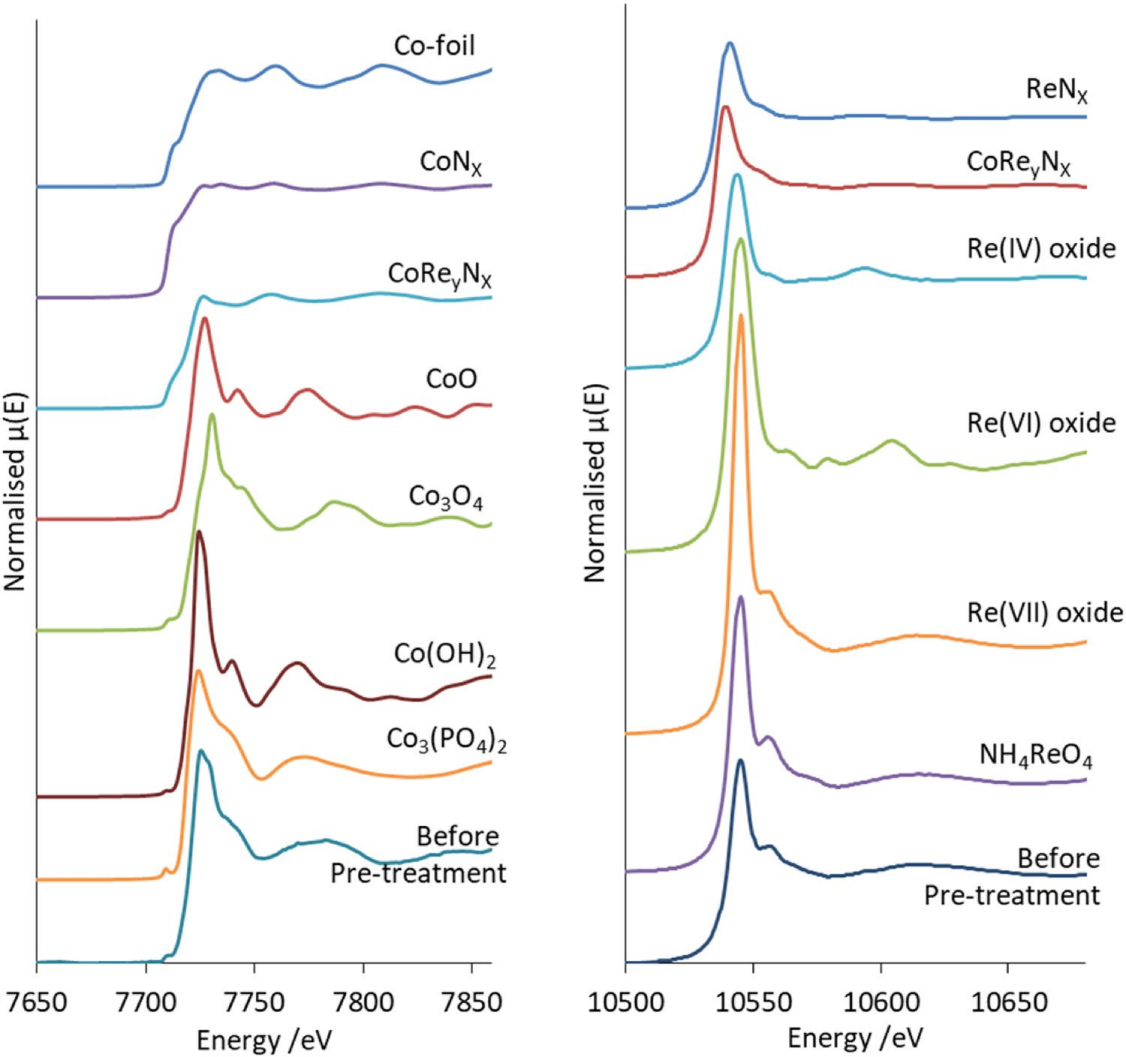
Normalised XANES for CoRe_1.6_ compared to reference compounds Co K-edge (left) and Re L-III edge (right)

Figure 1 shows the Co K-edge and Re L_III_-edge normalised XANES of the CoRe_1.6_ starting material compared to reference compounds. The fresh CoRe_1.6_ does not show similarities to the oxides and instead the local cobalt surrounding is more comparable to the cobalt phosphate reference. Comparing E_0_ values, it is clear cobalt is present as a Co(III)/Co(II) mixture in the fresh catalyst prior to pre-treatment. The XANES (Fig. 1) of the CoRe_1.6_ starting material resembles strongly the NH_4_ReO_4_ reference compound in also exhibiting similar absorption edge values to the Re(VII) references.

The EXAFS (Figure S.2) further confirms the above as refinements show an average coordination of four Co–O pairs at 1.96 Å. EXAFS refinements of the fresh CoRe_1.6_ yield 3.4 Re–O absorption pairs at 1.70 Å which is slightly shorter than those previously reported for tetrahedrally coordinated ReO_4_^−^ (1.74 Å in Bi/Re/O compounds), and longer than the Re=O double bonds reported from X-ray diffraction of organo-rhenium (VII) oxides (1.67 Å) [22]. By comparison the octahedral Re–O_Oh_ bond lengths are typically found at 1.89 Å [23]. A second Re–O shell with a multiplicity of 3 is found at 2.15 Å, which appears to be too long to be octahedrally coordinated ReO_6_^5−^. However similar Re–O bonds are also reported in the EXAFS studies of the oxides of Re/Bi mentioned above [23]. Hence it can be concluded that both cobalt and rhenium are present in a highly oxidised state in CoRe_1.6_ prior to pre-treatment (Table 1).

**Table 1:** Results from the least squares EXAFS analysis a at Re L-III-edge and Co K-edge for fresh CoRe4 (AFAC transferred from Co_3_O_4_ = 0.66 and NH_4_ReO_4_ = 0.67) and reference compounds CoRe_y_N_x_, CoN_X_ and ReN_x_ prepared by ammonolysis (AFAC transferred from Co-foil = 0.79 and Re-foil = 0.8)

Sample	Shell	N	R (Å)	2σ^2^ (Å^2^)	EF (eV)	R (%)	Δk
CoRe_1.6_	Co–O	4(1)	1.96(2)	0.003(9)	− 2(2)	58	2–8
	Re–O	3.4(3)	1.704(7)	0.003(1)	− 6(2)	28	2–13
	Re–O	3(1)	2.15(2)	0.02(1)			
CoRe_y_N_x_	Co–Co	4.6(7)	2.468(6)	0.018(3)	− 5.0(9)	29	2–10
	Co–Re	2.0(8)	2.66(1)	0.011(7)			
CoRe_y_N_x_	Re–N	0.4(2)	1.76(3)	0.01(1)	− 6(1)	16	3.5–9.5
	Re–Co	3.0(5)	2.57(1)	0.022(3)			
	Re–Re	3.1(8)	2.650(7)	0.018(5)			
CoN_x_	Co–Co	4.0(8)	2.50(1)	0.013(3)	− 8(2)	43	2–11
ReN_x_	Re–N	0.6(5)	1.79(3)	0.014(18)	3(2)	47	3.5–9
	Re–Re	7(2)	2.67(2)	0.032(8)			
Co-foil	Co–Co	12	2.496(7)	0.0132(8)	− 16(1)	33	2–12

^a^The EXAFS refinements give information about multiplicity (N), bonding distance (R) and thermal vibration (Debye–Waller factor, 2σ^2^). E_F_ is the refined correction of Fermi energy in vacuum, compared to E_0_. The standard deviation in the last significant digit as calculated by DL-excurv is given in parentheses. The deviation for 2σ^2^ is ± 20%.The Fit index is defined as ${\text{FI}}={\upsigma _i}(1/{\upsigma _i}){\left[ {{\text{Exp(i) }}-{\text{ Theory (i)}}} \right]^2}$. The statistical R-factor is defined as ${\text{R}}=\upsigma _{{\text{i}}}^{{\text{N}}}\left[ {1/{\upsigma _i}(|\upchi _{{\text{i}}}^{{{\text{exp}}}}(k){\text{ }} - \upchi _{{\text{i}}}^{{{\text{th}}}}(k)|)} \right] \times 100\%$ and gives indication of the quality of fit in k-space

### Denitridation

To obtain a first indication of the role of pre-treatment in the possible formation of an active CoRe_y_N_x_ nitride phase during the induction time, denitridation of N_2_/H_2_ pre-treated CoRe_1.6_ was undertaken using H_2_/Ar. Pre-treatment was undertaken using 75% H_2_ in N_2_ at 600 °C for 2 h. The subsequent H_2_/Ar reaction profile, presented in Figure S.3, displayed an overall drop in conductivity of 42 µS cm^− 1^ which corresponded to a total ammonia production of ~ 35 µmoles of ammonia over 200 min of reaction, after which no further ammonia production was observed. Table 2 displays observed rates of ammonia production for selected nitrides and also the equivalent cobalt rhenium material previously reported (which is denoted as CoRe_4_, representing its synthesis ratio) under comparable reaction conditions (400 °C, ambient pressure, 75% H_2_ in N_2_). The low amount of ammonia generated in the absence of dinitrogen gas feed suggests a very low content of N being stored within the CoRe_1.6_ system, and possibly results from residual N stored on the surface.

**Table 2:** Overview of ammonia synthesis rates for selected nitrides compared to denitridation of cobalt rhenium

Materials	Pre-treatment	Reaction conditions	Rate (µmol g^− 1^ h^− 1^)	Reference
Co_3_Mo_3_N	H_2_/N_2_	H_2_/N_2_	652	[4]
Ni_2_Mo_3_N	H_2_/N_2_	H_2_/N_2_	400	[1]
CoRe_4_	H_2_/N_2_	H_2_/N_2_	943	[9]
CoRe_4_	H_2_/Ar	H_2_/N_2_	844	[9]
CoRe_1.6_	H_2_/N_2_	H_2_/Ar	a	This paper

^a^Rate not determined, deactivation after 2 h of reaction

### Reducibility of the Cobalt Rhenium System

**Fig. 2:**
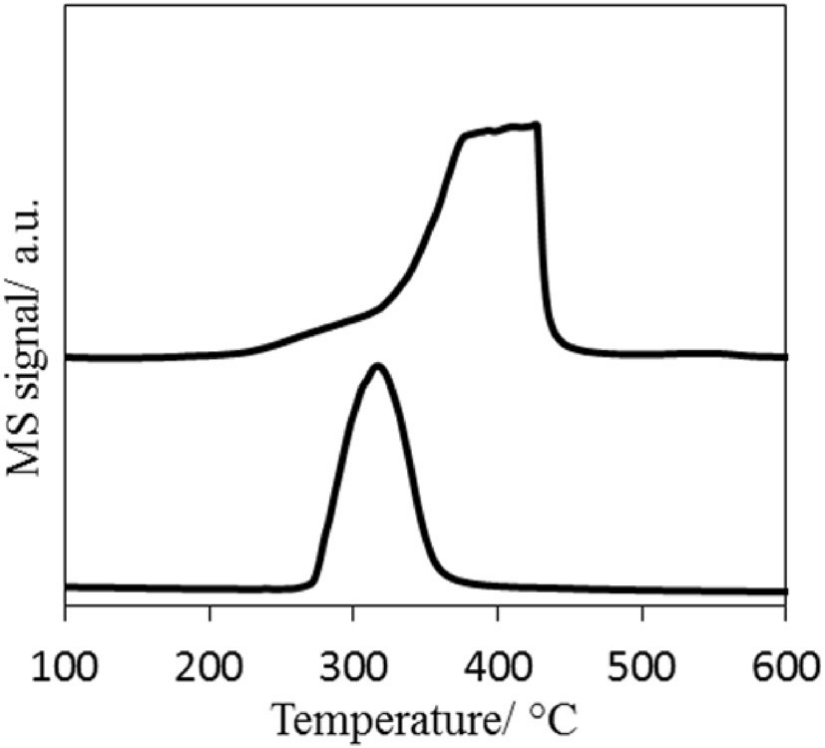
Temperature programmed reduction (TPR) profiles of CoRe_1.6_ catalyst (lower profile) and Co_2_O_3_ (upper profile) under H_2_ flow

The active form of the cobalt rhenium system is typically obtained after reduction during the pre-treatment step at high temperature. The reducibility of CoRe_1.6_ and Co_2_O_3_ was evaluated using H_2_ TPR (Fig. 2). The reduction of Co_2_O_3_ started at a temperature as low as 200 °C and was characterized by two reduction steps. A small peak around 230 °C can be observed which has been assigned in the literature to the reduction of Co^3+^ to Co^2+^ with the second peak occurring at higher temperature being attributed to the reduction of Co^2+^ to Co^0^ [24, 25]. The reduction profile of CoRe_1.6_ started at slightly higher temperature than the Co_2_O_3_ and was characterized by a single reduction step with a maximum located at 325 °C. Hydrogen spillover from rhenium to cobalt oxide is widely accepted to promote the reduction of cobalt oxide which could explain the profile reduction of the CoRe_1.6_ [26, 27].

### Effect of Different Pre-treatments; H_2_/Ar or H_2_/N_2_

**Fig. 3:**
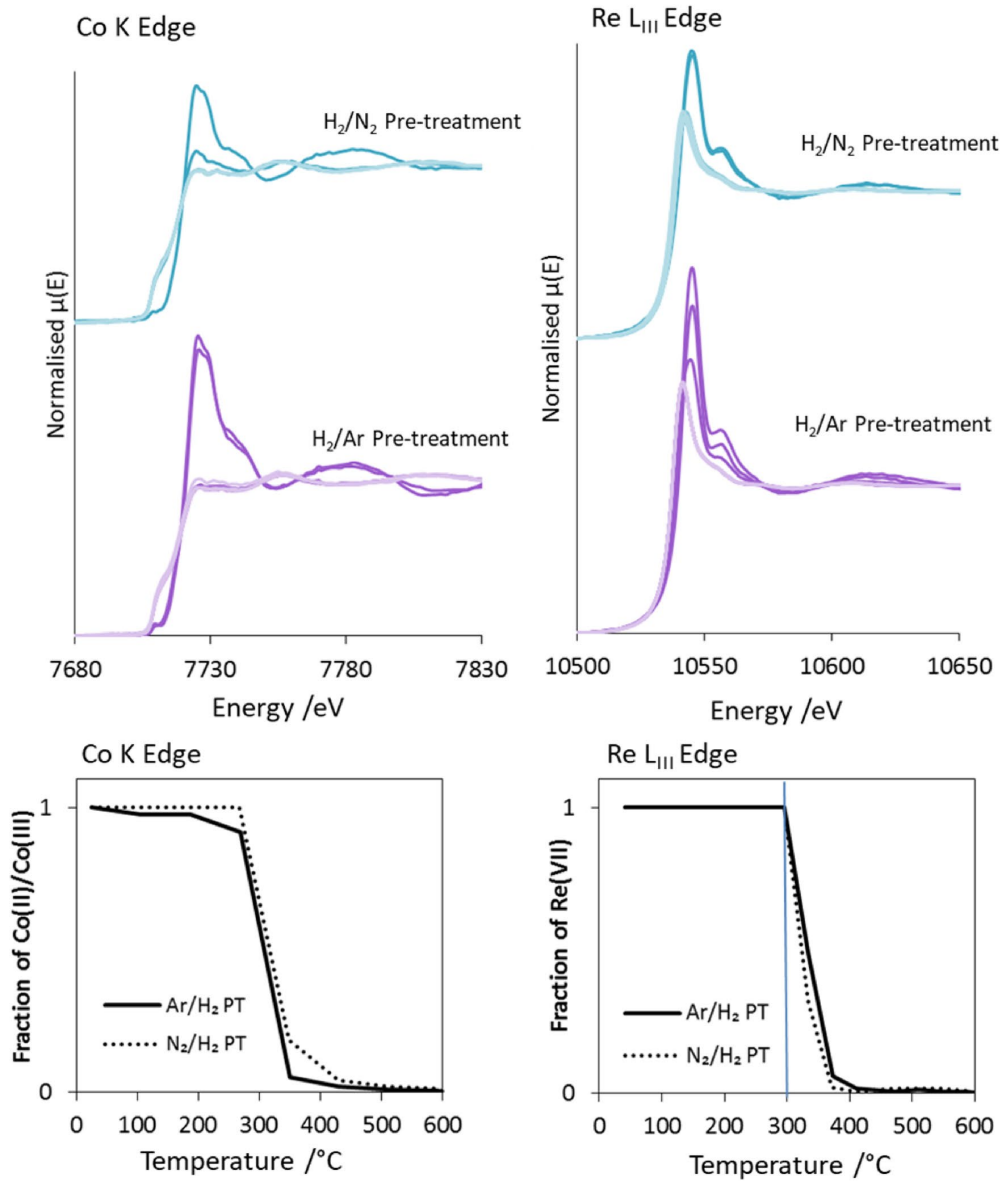
Selected normalized XANES (150 °C intervals) during pre-treatments in H_2_/Ar and H_2_/N_2_ XANES for Co K-edge (top left) and Re L_III_-edge (top right), and reduction profiles from linear combination of all normalized (30–150 eV) XANES in Athena (bottom)

Selected normalised XANES (at 150 °C intervals, including relevant changes) of CoRe_1.6_ during pre-treatment in 75% H_2_ in N_2_ and 75% H_2_ in Ar from ambient temperature to 600 °C are shown in Fig. 3. The figure also presents the reduction profiles determined from linear combination of all XANES spectra. The Co^III/II^→Co^0^ reduction occurs quite steeply, is initiated around 200 °C, and clearly occurs before the reduction of Re^VII^→Re^0^, which starts at 300 °C for both pre-treatments as seen in Fig. 3. The starting point for the first reduction step for cobalt is somewhat lower for the H_2_/Ar pre-treatment. Typically complete reduction of rhenium requires much higher temperatures [28], hence these results suggest a cooperative effect between rhenium and cobalt, similar to the hydrogen spillover effect which has been reported for the Pt–Re system [29]. Both elements are reduced to the metallic state between 500 and 600 °C. Other studies have also suggested the role of rhenium being to be the promotion of reduction of cobalt [15]. However, in the current study it is clear that cobalt initiates reduction of rhenium in the CoRe_1.6_ during both H_2_/Ar and H_2_/N_2_ pre-treatments.

**Fig. 4:**
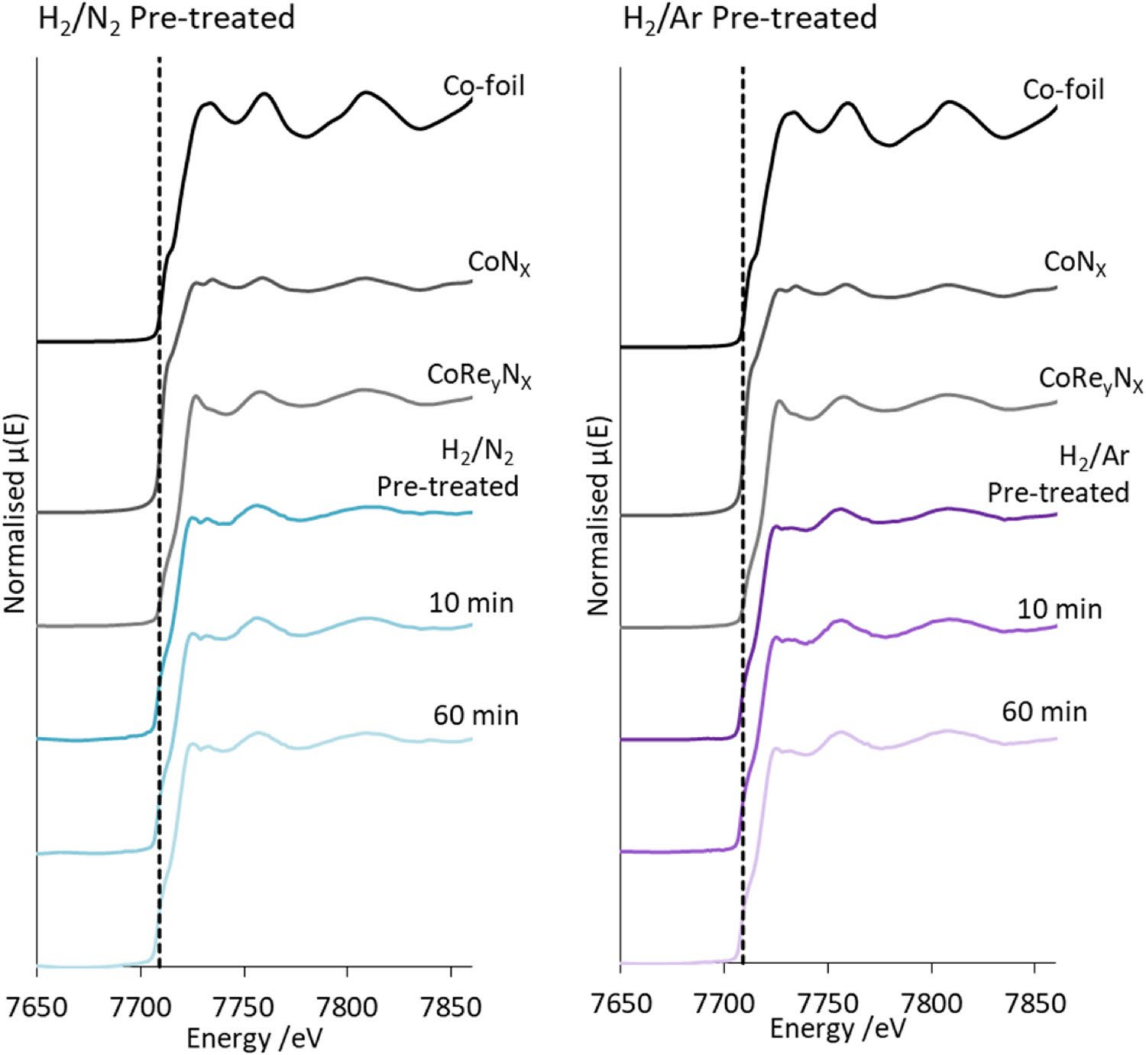
Normalised XANES for CoRe_1.6_ during ammonia synthesis compared to reference compounds for Co K-edge for H_2_/N_2_ pre-treated (left) and H_2_/Ar pre-treated (right)

**Fig. 5:**
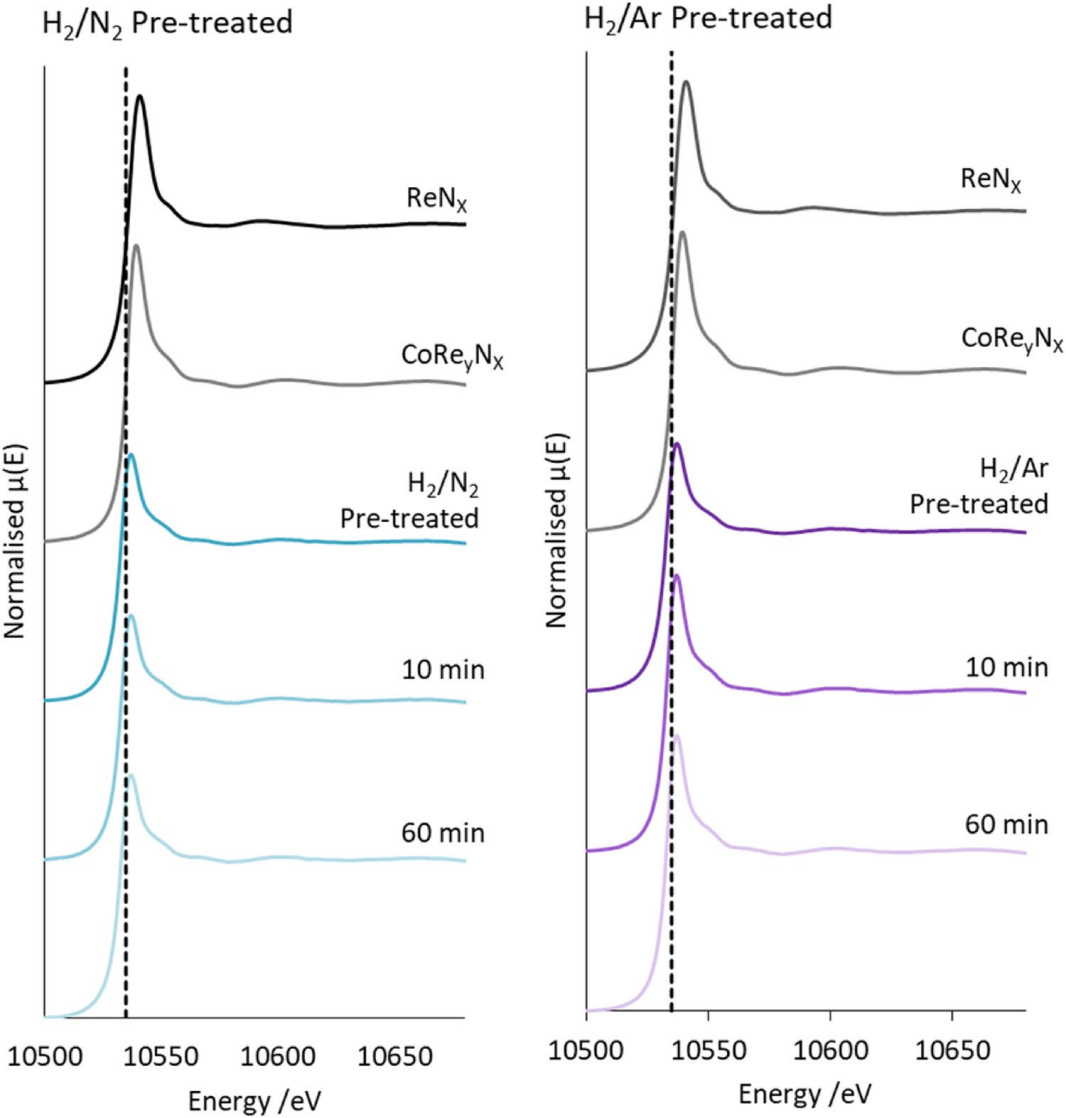
Normalised XANES for CoRe_1.6_ during ammonia synthesis compared to reference compounds for Re L-III edge for H_2_/N_2_ pre-treated (left) and H_2_/Ar pre-treated (right)

The normalised XANES of CoRe_1.6_ after the two pre-treatments compared to references at the Co K-edge and Re L_III_-edge are shown in Figs. 4 and 5 respectively. Both the shape of the XANES and the obtained E_0_ values confirm that the metals are present in the metallic state, however the cobalt data show two small features in the white line region (Fig. 4), whereas the cobalt foil shows one broad feature. These two smaller white line features are also reported in the cobalt XANES for the PtCo/Al_2_O_3_ system after in situ H_2_ reduction, hence it would seem indicative of bimetallic Co–M interaction [30]. We cannot rule out re-oxidation in air of the post-ammonolysis samples as they were collected ex situ. The normalised rhenium XANES for the CoRe_1.6_ clearly appear at a lower E_0_ value also displaying a lower white line intensity (Fig. 4) when compared to CoRe_y_N_x_ and ReN_x_ prepared by ammonolysis. This would confirm that rhenium in CoRe_1.6_ is fully reduced, whereas rhenium is partially re-oxidised in the post-ammonolysis references. There are clear similarities between the features observed in the XANES independent of pre-treatment for both edges, indicating that there are largely similar local metal surroundings at this point.

For the EXAFS analysis, attention was directed towards analysis of the structure of the cobalt rhenium phase and more precisely the mixing and bimetallic nature of the material during the pre-treatment and ammonia synthesis. The degree of bimetallic mixing is obtained by comparing the obtained average coordination of the first absorption shell which for the cobalt edge will comprise Co–Co and/or Co–Re, and similarly Re–Re and/or Re–Co at the rhenium edge. As EXAFS sees sums of contributions both absorption pairs will be present on respective edges if the material consists of a mixture of mono- and bimetallic phases. However, as these shells exhibit different bond distances the contributions should be resolved in EXAFS refinements. In a bimetallic system the degree of mixing is often best obtained from the minority element, which in our case is Co [31]. This can be exemplified by the following relationship C_Re_ × N_Re−Co_ = C_Co_ × N_Co−Re_, where C_Re_ and C_Co_ are the concentrations of each metal, which means that heteroatomic bonds will be better visualized at the element of low concentration in bimetallic phases [30]. However, due to rhenium being a heavy absorber optimising the dilution for XAS data collection was challenging, which in turn led to in situ cobalt data being of reduced quality. Another challenge in this study is obtaining reliable information about rhenium as a backscatterer due to the fact that a large part of the EXAFS signal lies in the high k-region, and the data in this study utilises a k-window of 3.5–9.5 Å^−1^ as the resolution beyond this value is limited by the in situ conditions.

Results from EXAFS analysis of references prepared by ammonolysis (CoRe_y_N_x_, CoN_x_ and ReN_x_) are shown in Table 1 and Figure S.2 confirming that the process has led to the reduction to the metallic state in all three samples. Bimetallic mixing is confirmed for CoRe_y_N_x_ where coordination numbers of 4.6 for Co–Co and 2.0 for Co–Re are found at the cobalt edge, while average coordination numbers at the rhenium edge are 3.1 for Re–Re and 3.0 for Re–Co. The bond-distance obtained for the Co–Co bond in CoRe_y_N_x_ is 2.47 Å, which is slightly shorter than that obtained for the Co-foil (2.49 Å), and the Re–Re bond length is 2.65 Å, which is shorter than that of Re-foil (2.74 Å). From refinements, the Co–Re bond length is 2.66 Å, while the corresponding Re–Co bond length at the rhenium edge is 2.57 Å. Shorter Re–Re bonds were also reported by Rønning et al. for the Co–Re/Al_2_O_3_ system where, similar to our findings, they found the Re–Co bond length to be 2.54 Å [15]. These numbers do in fact confirm bimetallic Co–Re mixing occurs in the sample, however as relatively high multiplicity Co–Co and Re–Re absorption pairs are found during refinements, one cannot exclude the possibility that pure Co and Re phases occur in the sample. Nitride formation, suggested by the partially oxidised rhenium from XANES for this sample, is confirmed by a Re–N shell at 1.76 Å. The low average Re–N multiplicity of 0.4 and high Re–Re/Co coordination suggests there is still a large degree of metallic rhenium in this sample. A Re–N shell with average coordination of 0.6 is also found in the ReN_x_ sample at 1.79 Å, in addition to the Re–Re shell at 2.67 Å. For the CoN_x_ sample, only the Co–Co shell at 2.50 Å with an average coordination of 4 is found in the refinement.

**Fig. 6:**
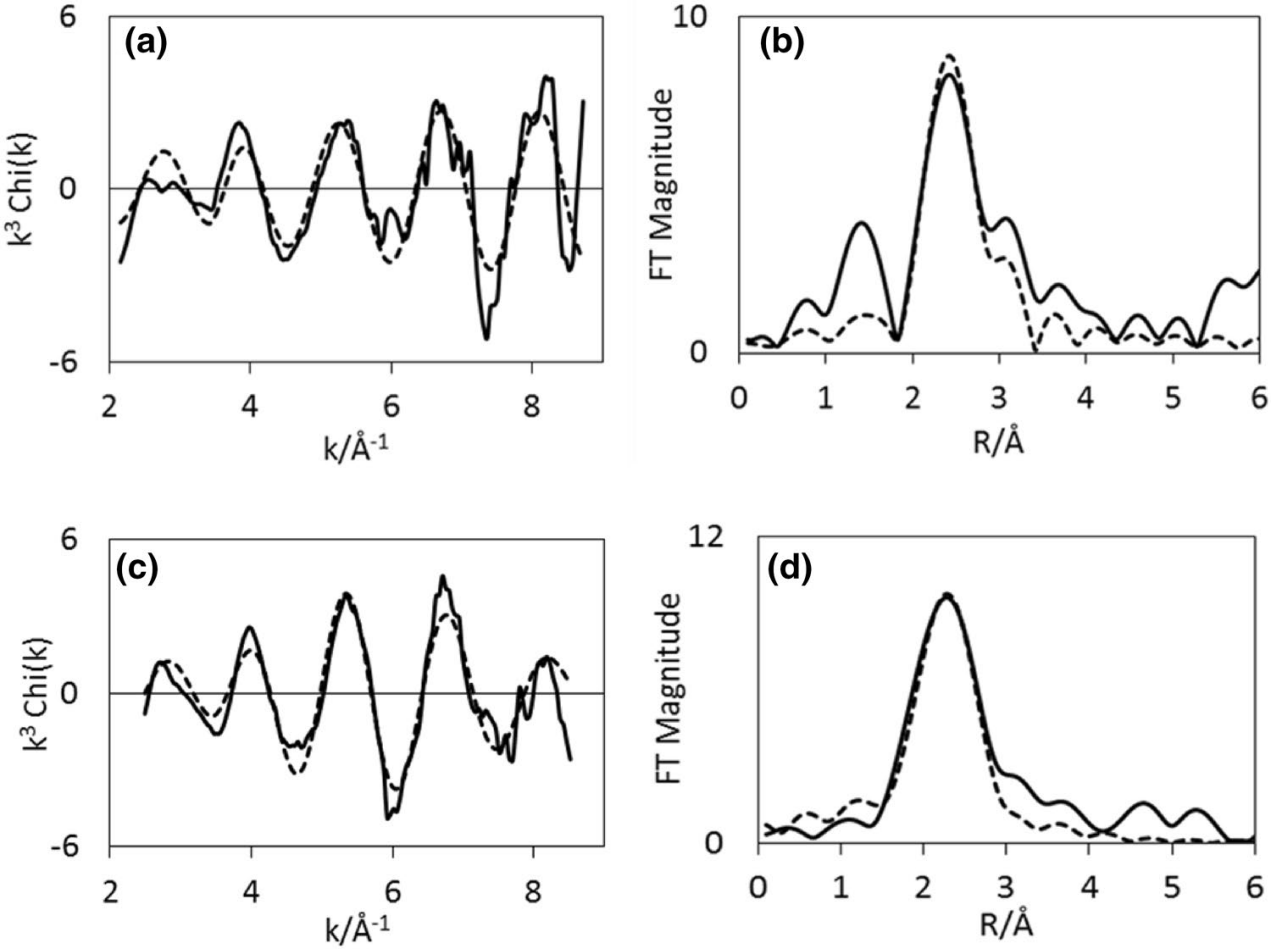
Experimental (line) and calculated (dotted line) k^3^-weighted EXAFS (left) and its Fourier Transform (right) for CoRe_1.6_ after pre-treatment in H_2_/N_2_ (**a**–**b**) and H_2_/Ar (**c**–**d**) for the Co K-edge

**Fig. 7:**
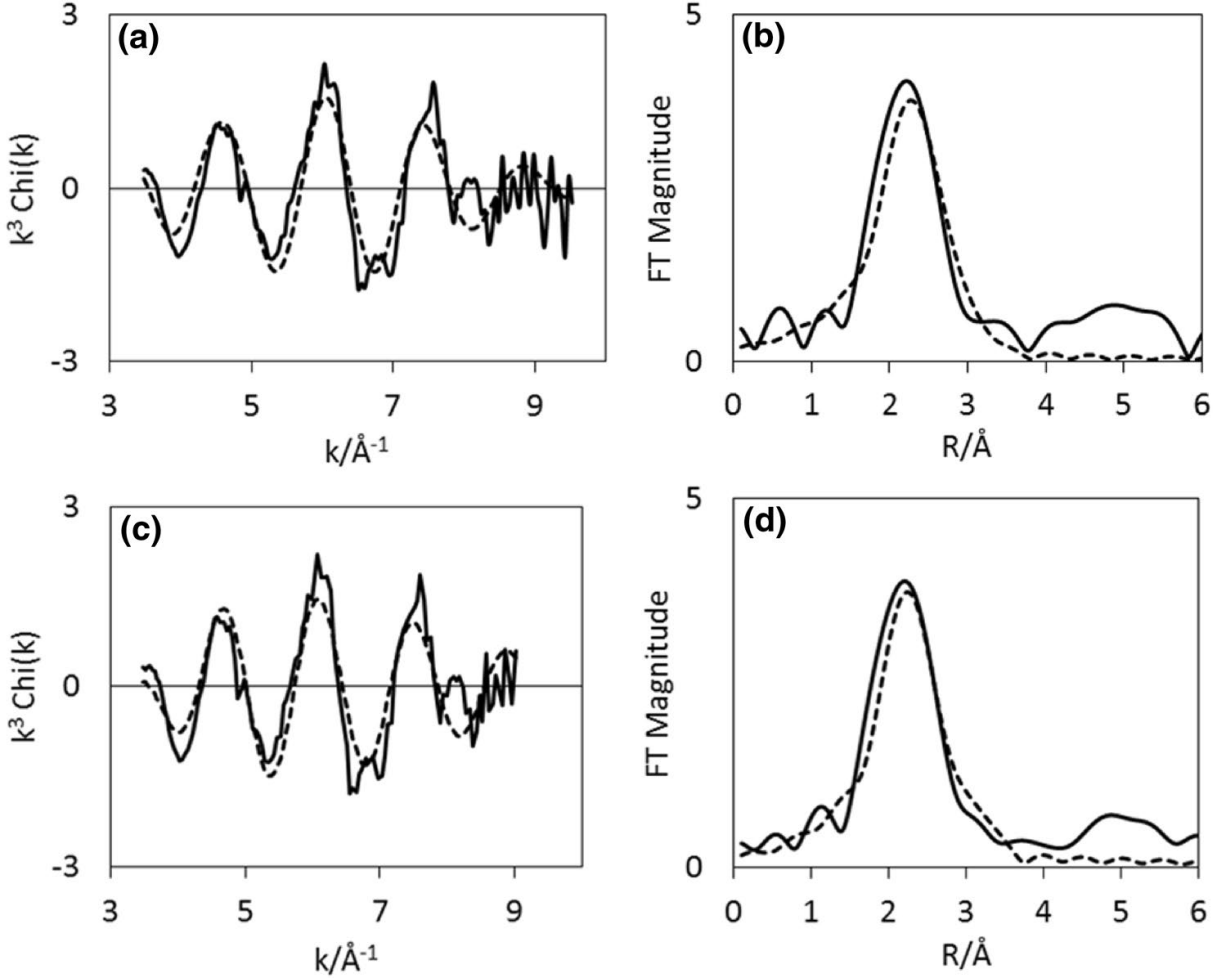
Experimental (line) and calculated (dotted line) k^3^-weighted EXAFS (left) and its Fourier Transform (right) for CoRe_1.6_ after pre-treatment in H_2_/N_2_ (**a**–**b**) and H_2_/Ar (**c**–**d**) for the Re-L-III edge

EXAFS of CoRe_1.6_ after the two different pre-treatments clearly confirms that the presence of nitrogen during pre-treatment is a prerequisite for extensive bimetallic Co–Re mixing in CoRe_1.6_ (Table 3). While there are clear similarities in the EXAFS and Fourier Transform spectra at the rhenium edge (Fig. 7), there are clear differences in the cobalt EXAFS (Fig. 6). Cobalt, being the minor constituent in the sample, is the element which would be more sensitive to the degree of mixing. A large degree of Co–Re bimetallic mixing is observed in CoRe_1.6_ after pre-treatment in 75% H_2_ in N_2_, yielding coordination numbers of 3.2 for Co–Re and 3 for Re–Co at 2.51 Å. As observed for the post-ammonolysis samples, CoRe_1.6_ also exhibits Co–Co (1.7, at 2.45 Å) and Re–Re (5, at 2.71 Å) backscattering pairs, suggesting pure cobalt and rhenium phases are also present. It is worth noting that the lack of second or third metal–metal scattering shells in these samples indicates a high degree of disorder in the system. Typically a second Re-metal distance is found at 3.56 Å in bulk rhenium, which is absent in the CoRe_1.6_ system. This again is reflected in the XRD patterns (Figure S.5), which show a degree of amorphous background. It should be noted that attempts to introduce a Re–N shell at low R-value led to a decrease in fit-factor. Reasonable fit-values could not be obtained and the shell was therefore omitted.

**Table 3:** EXAFS least squares refinements of CoRe_1.6_ after 2 h pre-treatment at 600 °C in either H_2_/N_2_ or H_2_/Ar and during ammonia synthesis at 400 °C for both Co K-edge and Re L_III_-edge from in situ XAS (AFAC transferred from Co-foil = 0.79 and Re-foil = 0.8)

Sample	Shell	N	R (Å)	2σ^2^ (Å^2^)	E_F_ (eV)	R (%)	Δk
CoRe_1.6_ H_2_/N_2_ 600 °C	Co–Co	1.7(6)	2.45(1)	0.011(7)	− 6.3(8)	39	2–8.5
	Co–Re	3.2(9)	2.51(1)	0.020(6)			
	Re–Co	3(1)	2.51(2)	0.042(9)	− 7(2)	49	3.5–9.5
	Re–Re	5(3)	2.71(3)	0.05(2)			
CoRe_1.6_ H_2_/Ar 600 °C	Co–Co	9(2)	2.46(1)	0.049(5)	− 3(1)	36	2–8.5
	Re–Co	1.7(8)	2.51(1)	0.031(9)	− 9(1)	47	3.5–9.5
	Re–Re	7(3)	2.73(1)	0.05(1)			
H_2_/N_2_ PT 10 min	Co–Co	5(1)	2.47(1)	0.029(5)	− 6.1(8)	29	2–8.5
	Co–Re	2.1(7)	2.52(1)	0.010(7)			
	Re–Co	3(2)	2.54(6)	0.05(2)	− 7(2)	45	3.5–9.5
	Re–Re	4(1)	2.66(1)	0.027(7)			
H_2_/N_2_ PT 60 min	Co–Co	2.7(8)	2.509(7)	0.013(7)	− 4.0(8)	37	2–8.5
	Co–Re	2.7(7)	2.486(8)	0.005(5)			
	Re–Co	0.9(6)	2.48(1)	0.013(9)	− 8(2)	45	3.5–9.5
	Re–Re	5(2)	2.70(1)	0.029(8)			
H_2_/Ar PT 10 min	Co–Co	5.4(9)	2.460(9)	0.026(4)	− 1(1)	29	2–8.5
	Re–Co	1.2(5)	2.56(2)	0.013(6)	− 9(1)	42	3.5–9.5
	Re–Re	5(2)	2.68(1)	0.030(8)			
H_2_/Ar PT mins	Co–Co	4.7(8)	2.452(8)	0.024(4)	− 1(1)	29	2–8.5
	Co–Re	2(2)	2.62(8)	0.07(5)			
	Re–Co	0.7(3)	2.531(9)	0.002(6)	− 11(1)	36	3.5–9.5
	Re–Re	5(2)	2.743(9)	0.026(6)			

PT refers to pre-treatment and the times given correspond to reaction times under ammonia synthesis conditions

**Fig. 8:**
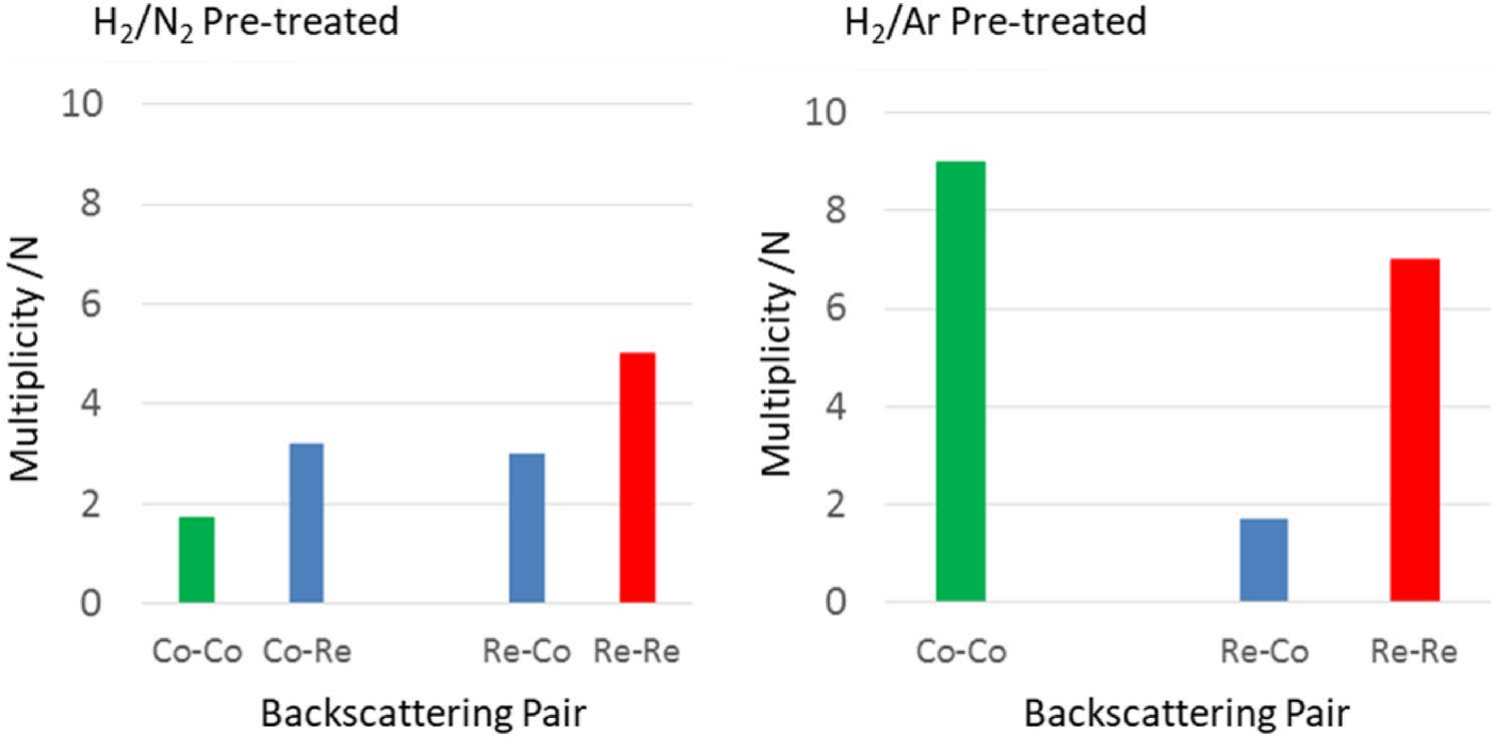
Average coordination numbers (N) from EXAFS analysis for CoRe_1.6_ after 120 min pre-treatment at 600 °C in H_2_/N_2_ (left) or H_2_/Ar (right) for Co K-edge and Re-L_III_ edge

For CoRe_1.6_ pre-treated in 75% H_2_ in Ar, attempts to introduce a Co–Re coordination shell were unsuccessful, yielding only a Co–Co shell at 2.46 Å with average coordination number of 9. At the rhenium edge, a Re–Co shell could be fitted, with average coordination of 1.7 at 2.51 Å. The major contribution to the signal is the Re–Re shell at 2.73 Å with average coordination number of 7. Clearly, the degree of mixing is greatly influenced by the pre-treatment, as the presence of nitrogen ensures bimetallic Co–Re pair formation. Average coordination numbers for the two pre-treatments of CoRe_1.6_ are displayed in Fig. 8.

### Structural Changes During Ammonia Synthesis

**Fig. 9:**
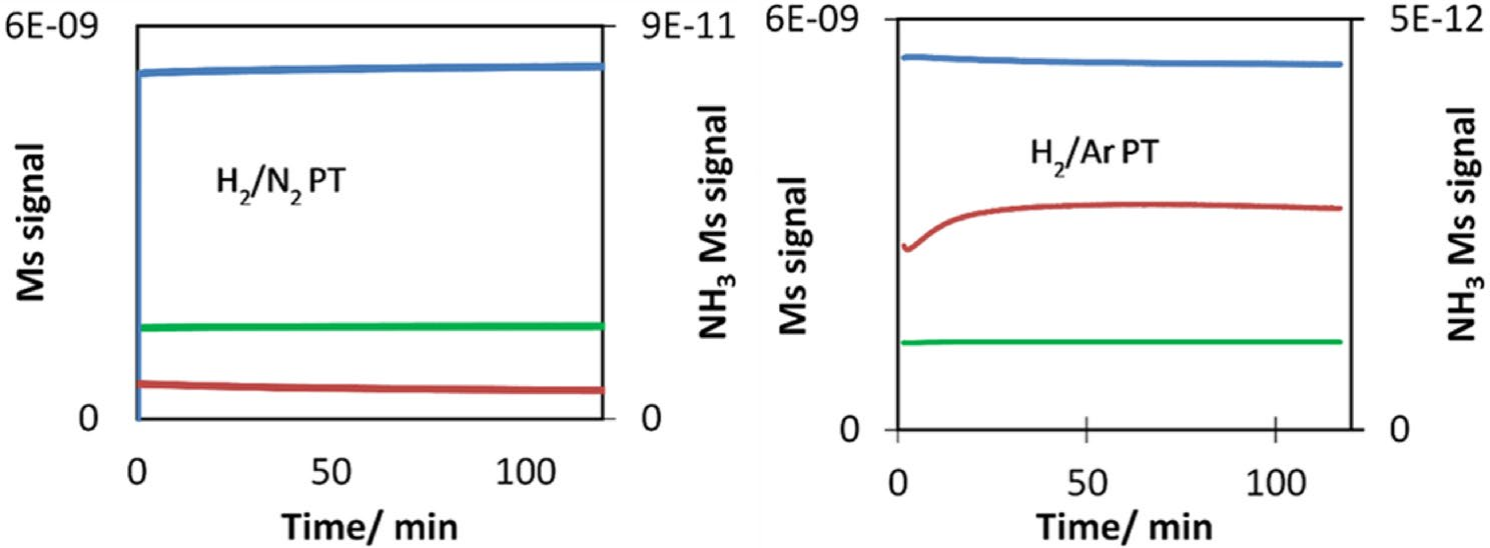
Online ion current signals showing ammonia production over time (NH_3_ = red, N_2_ = green and H_2_ = blue) from mass spectrometry during in situ XAS/XRD data collection for CoRe_1.6_ at 400 °C in 75% H_2_ in N_2_ reaction gas. The ammonia signal confirms the occurrence of a lag-time following H_2_/Ar pre-treatment as observed during catalytic measurements

Following pre-treatment, CoRe_1.6_ was cooled to 400 °C where ammonia synthesis was carried out for 2 h in 75% H_2_ in N_2_. The mass spectrometer (MS) signal is shown in Fig. 9, confirming the 20 min lag-time before ammonia production starts for the sample pre-treated in H_2_/Ar, as has already been reported [9]. For CoRe_1.6_ pre-treated in H_2_/N_2_ (reaction gas) ammonia production starts immediately. At this stage, as we have already established that the two pre-treatments affect the degree of Co–Re mixing and it is of interest to establish whether the observed lag-time can be explained by structural changes observable in in situ XAS. Following pre-treatment, CoRe_1.6_ was cooled to 400 °C where ammonia synthesis was carried out for 2 h in 75% H_2_ in N_2_.

The normalised XANES at both edges taken after 10 and 60 min under ammonia synthesis reaction conditions are displayed in Figs. 4 and 5. No changes appear to occur in the cobalt XANES when compared to the spectra after the pre-treatment. The XANES are also similar independently of pre-treatment. Interestingly, when examining the rhenium normalised XANES, it is clear that the white line of the CoRe_1.6_ sample pre-treated in H_2_/Ar shows an increase in intensity 10 and 60 min into ammonia synthesis. The intensity of the white line for Re L_III_-edge XANES is caused by the transition from 2*p*_3/2_ to 5*d*, and typically the higher the oxidation state, the higher the white line. It is often regarded as difficult to extract quantitative information from variations in white line intensity for the rhenium L_III_-edge. However it seems clear that the observed change in electronic state of the Re species is not correlated with a similar change in the Co species. Hence, the change seems to occur in a pure Re-phase, and corresponds to the material becoming active for ammonia synthesis. The observed increase in intensity could possibly be an effect of partial oxidation of rhenium. However attempts to fit a Re–N shell for this sample were not successful.

**Fig. 10:**
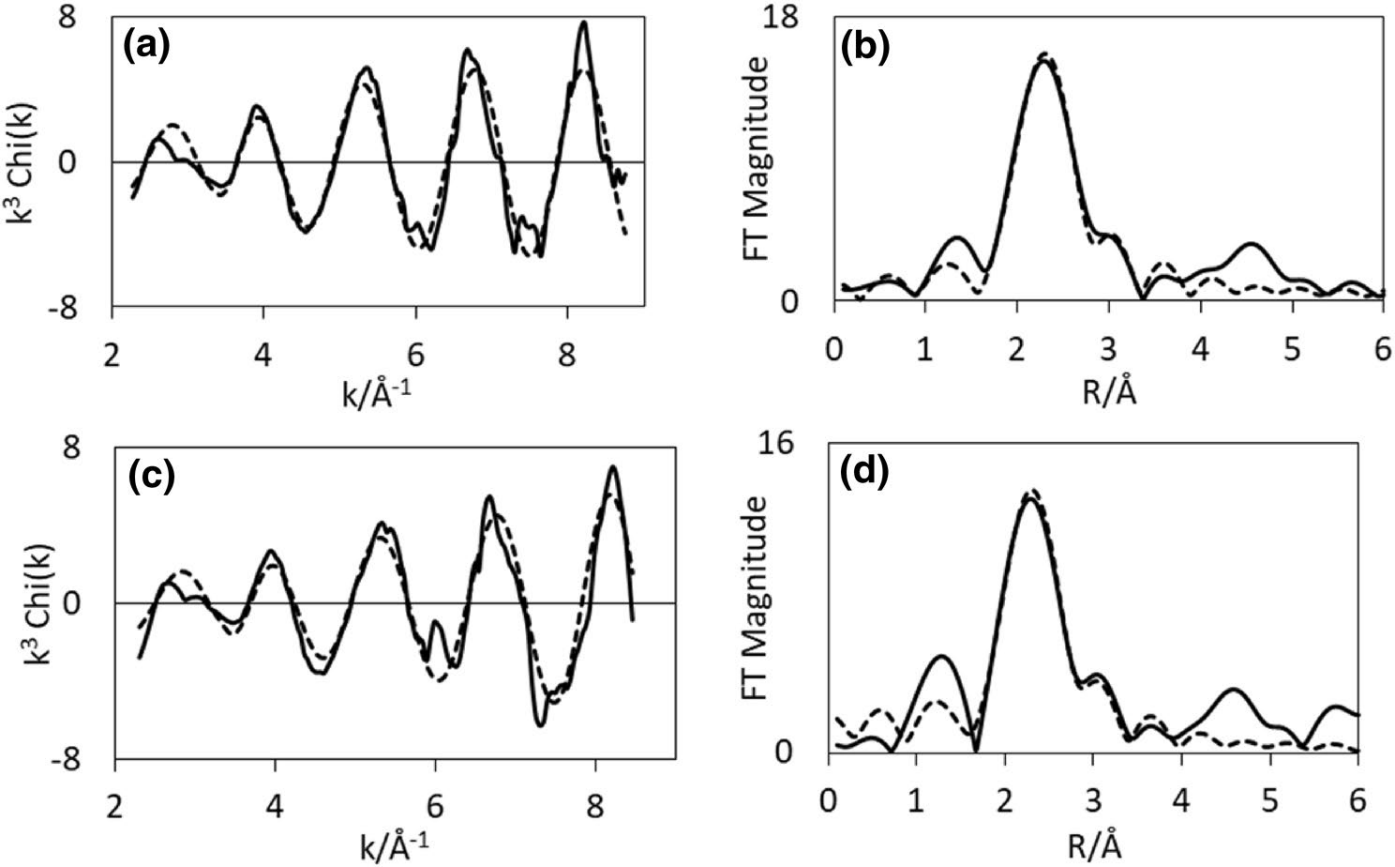
Experimental (line) and calculated (dotted line) k^3^-weighted EXAFS (left) and its Fourier Transform (right) for CoRe_1.6_ after pre-treatment in H_2_/N_2_ and after time on stream 10 min (**a**–**b**) and 60 min (**c**–**d**) in H_2_/N_2_ at 400 °C for the Co K-edge

**Fig. 11:**
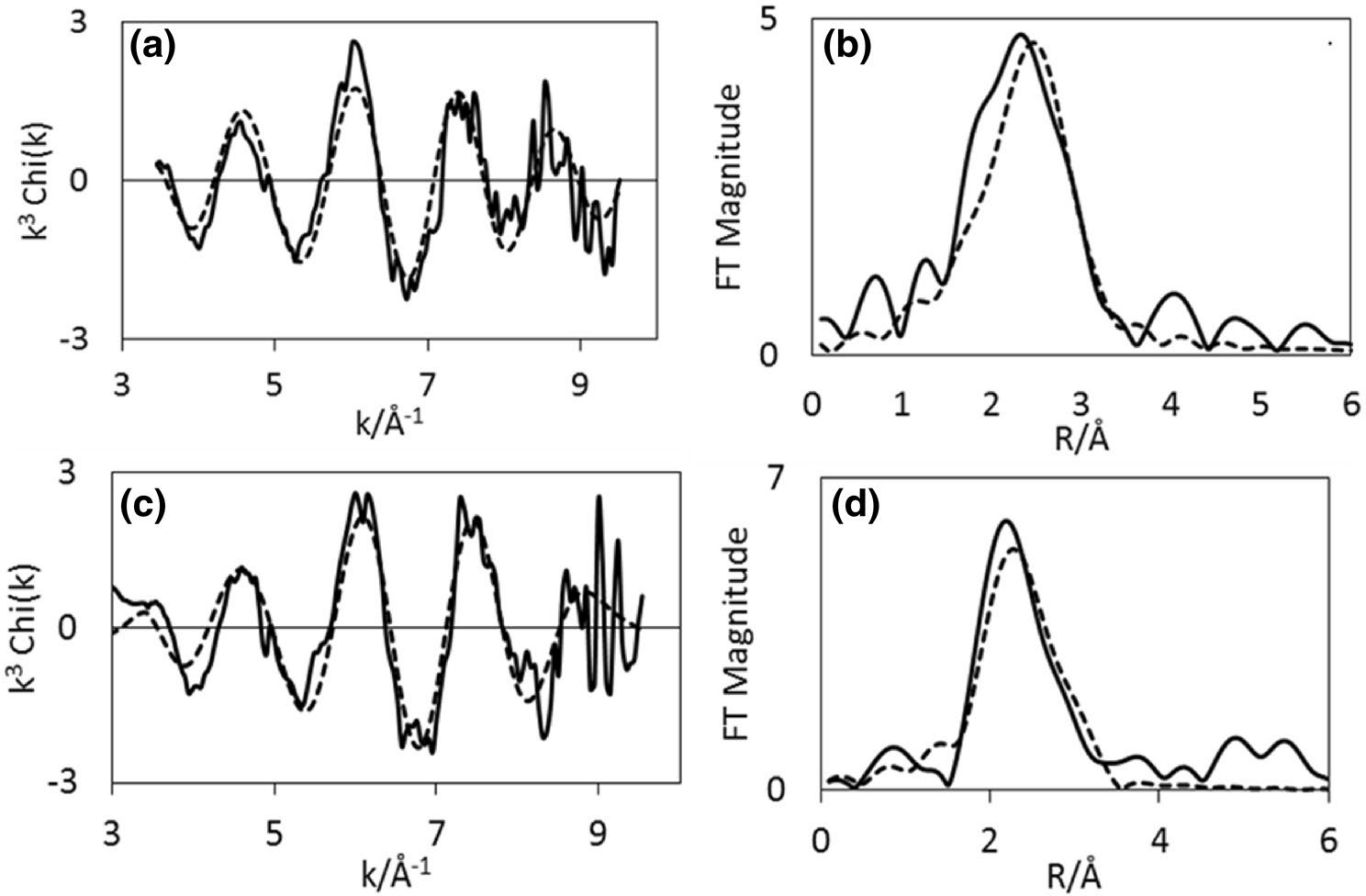
Experimental (line) and calculated (dotted line) k^3^-weighted EXAFS (left) and its Fourier Transform (right) for CoRe_1.6_ after pre-treatment in H_2_/N_2_ and after time on stream 10 min (**a**–**b**) and 60 min (**c**–**d**) in H_2_/N_2_ at 400 °C for the Re L_III_-edge

Results from EXAFS refinements (Table 3), show that the Co–Re mixing observed in the sample pre-treated in H_2_/N_2_ also remains during ammonia synthesis at 400 °C. After 10 min 2.1 Co–Re distances are fitted at 2.52 Å, and corresponding three Re–Co distances are found at 2.54 Å at the rhenium edge. After 60 min, 2.7 Co–Re distances remain at 2.48 Å, but at the rhenium edge only 0.9 is found at 2.48 Å. This could suggest some reorganisation of the nanostructure of the material and possible segregation of species occurs during reaction. There is a small shortening of the Co–Re/Re–Co average distance. During time on stream, Co–Co is reduced from 5 to 2.7 in multiplicity, and this shell undergoes an elongation from 2.47 to 2.51 Å. Similarly, the Re–Re bond is slightly elongated from 2.66  to 2.70 Å, but remains at 4–5 in average coordination number during the process. Experimental and calculated EXAFS and Fourier Transforms for CoRe_1.6_ following pre-treatment in H_2_/N_2_ after 10 and 60 min of reaction time show only minor changes (Figs. 10, 11).

**Fig. 12:**
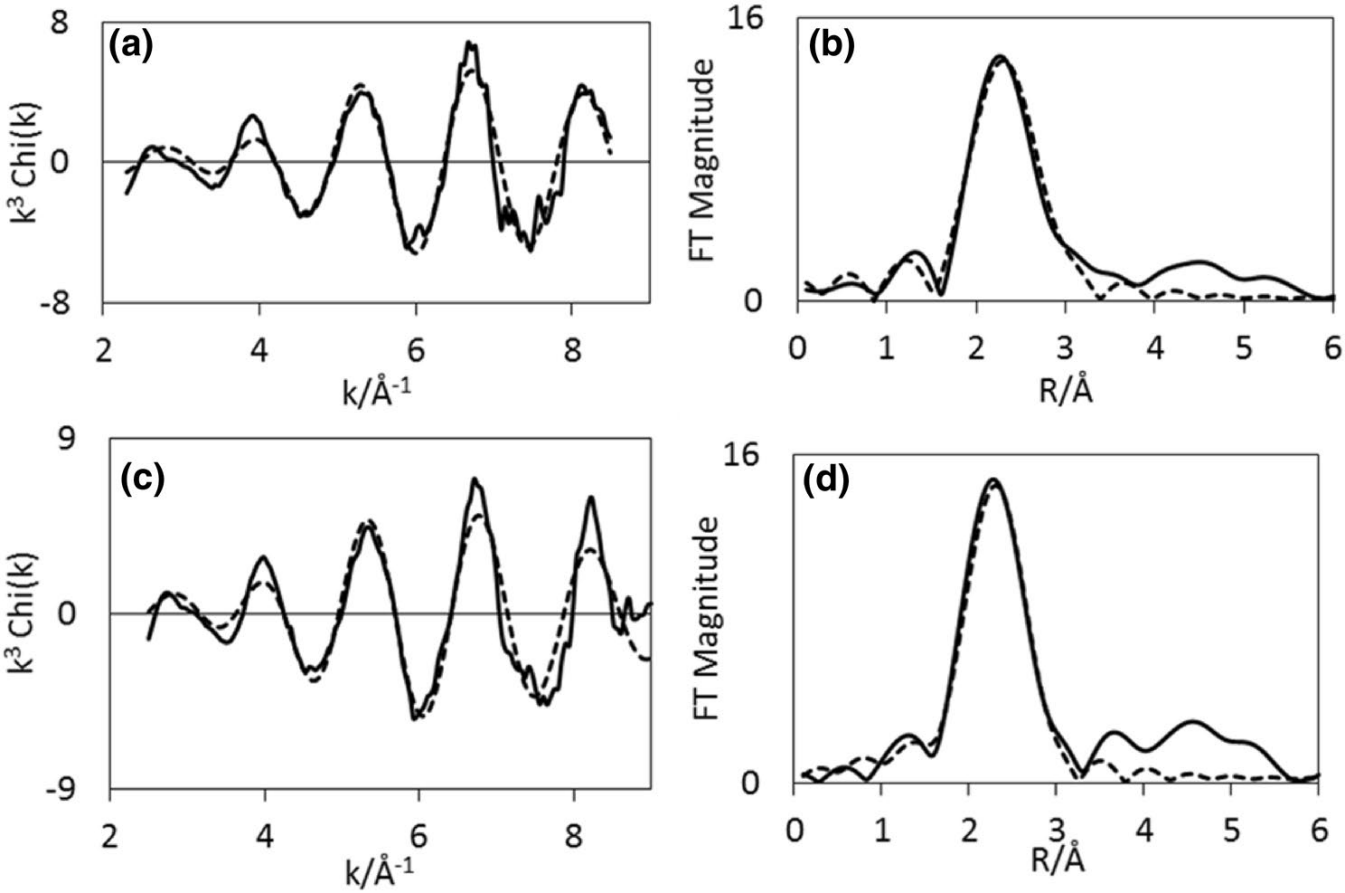
Experimental (line) and calculated (dotted line) k^3^-weighted EXAFS (left) and its Fourier Transform (right) for CoRe_1.6_ after pre-treatment in H_2_/Ar and after time on stream 10 min (**a**–**b**) and 60 min (**c**–**d**) in H_2_/N_2_ at 400 °C at the Co K-edge

**Fig. 13:**
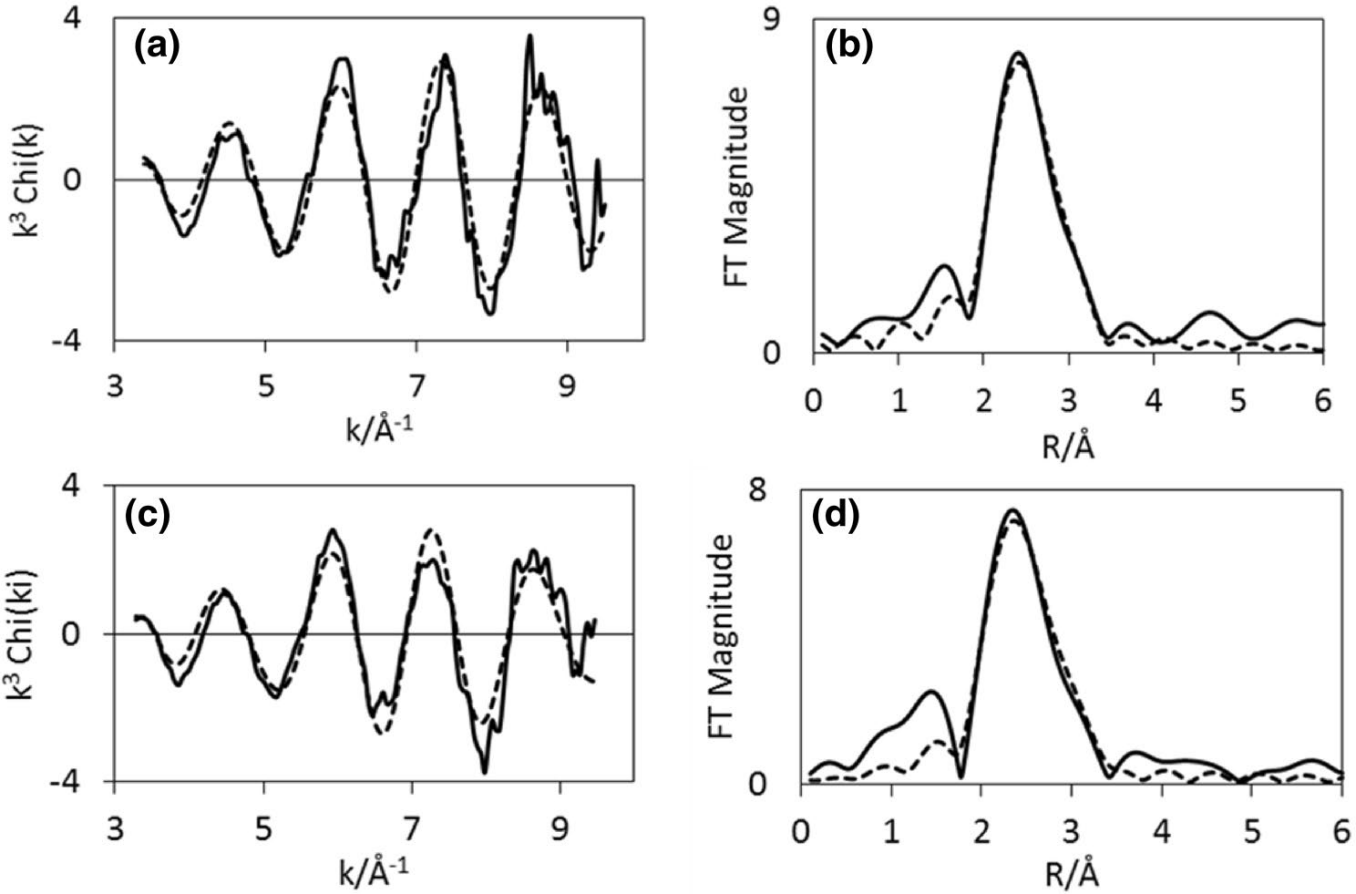
Experimental (line) and calculated (dotted line) k^3^-weighted EXAFS (left) and its Fourier Transform (right) for CoRe_1.6_ after pre-treatment in H_2_/Ar and after time on stream 10 min (**a**–**b**) and 60 min (**c**–**d**) in H_2_/N_2_ at 400 °C at the Re L_III_-edge

**Fig. 14:**
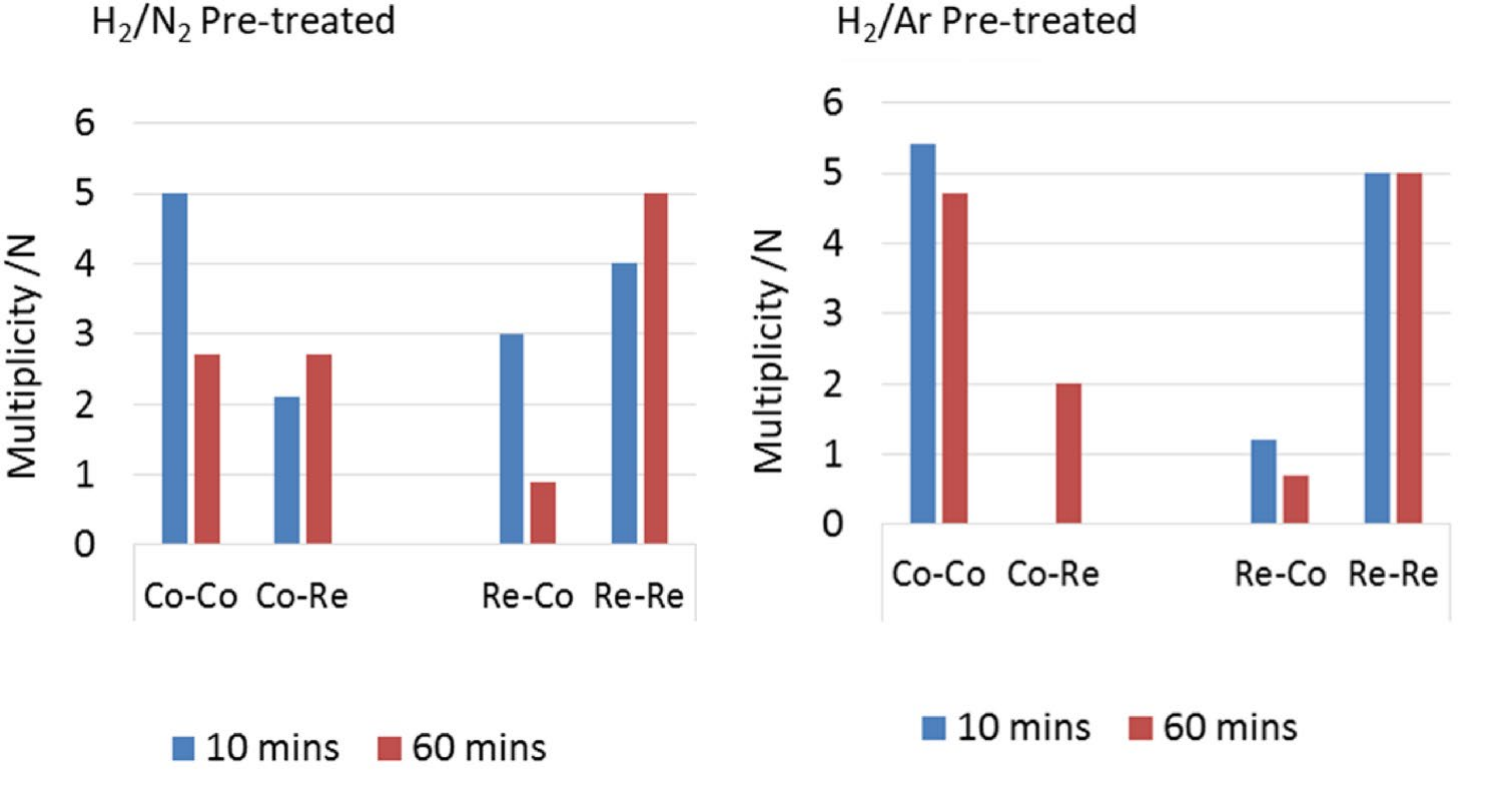
Average coordination numbers (N) from EXAFS analysis for CoRe_1.6_ during ammonia synthesis after pre-treatment at 600 °C in H_2_/N_2_ (left) or H_2_/Ar (right) at the Co K-edge and the Re L_III_-edge

By comparison, experimental and calculated EXAFS spectra of the sample pre-treated in H_2_/Ar are shown in Figs. 12 and 13, and while there appears to be only small changes at the cobalt edge, the changes in the local rhenium surroundings are more apparent. The lack of Co–Re mixing is still present following the pre-treatment in H_2_/Ar and during subsequent H_2_/N_2_ reaction at 400 °C, with only 5.4 Co–Co distances being fitted at 2.46 Å after 10 min of reaction at the cobalt edge (Fig. 14). After 60 min, the 4.6 Co–Co distances remain at 2.45 Å, but adding a second Co–Re contribution at 2.62 Å with average coordination of 2 improves the fit. However, the uncertainties and specifically the Debye–Waller factor are questionable suggesting the major contribution is still in fact only Co–Co. At the rhenium edge, the major contribution is still Re–Re with average coordination of 5 during reaction, although the bond elongates from 2.68 to 2.73 Å. A low coordinated (1.2 and 0.7) Re–Co interaction remains at 2.56–2.53 Å during the reaction. Refinements reveal the Co–Re mixing to be irreversible, but as the Re–Co shell is present, some mixing is obtained also after pre-treatment in H_2_/Ar. A comparison of the Fourier Transformed EXAFS spectra for the pre-treatment and time on stream is shown in Figures S.6 and S.7.

## Discussion

### Effect of Pre-treatment on the Phases Formed in Cobalt Rhenium

**Fig. 15:**
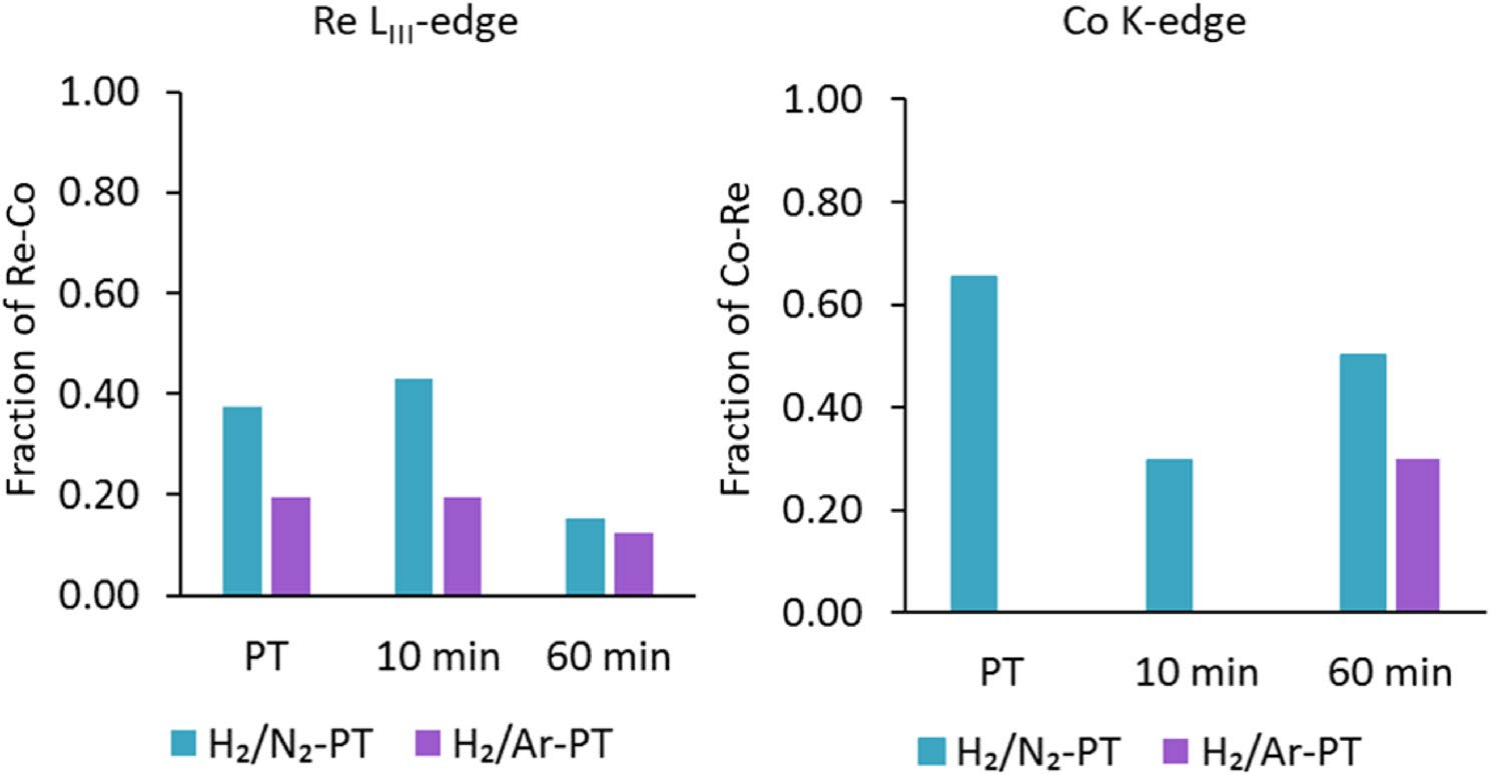
The fraction of bimetallic phase determined at the Re L_III_-edge and Co K-edge

The in situ XAS results can be summarised as follows; the presence of Co–Co, Co–Re and Re–Re interatomic distances indicate there to be a mixture of phases in the CoRe_1.6_ system during ammonia synthesis, with both Co–Re bimetallic species, and monometallic Re and Co-species being evident. The lack of a second metal–metal absorption shell in the analysis of both Co K-edge and Re L_III_-edge EXAFS indicate a high degree of disorder in the major phase. The fraction of bimetallic phase has been calculated after the method of Shibata et al. [32] based on the average coordination number (N) of Re–Co and Co–Re shells and the total coordination number (N_Re–Re_ + N_Re–Co_, N_Co–Co_ + N_Co–Re_) to follow the amount of bimetallic phase, as shown in Fig. 15. After pre-treatment in H_2/_N_2_, the major contribution is the Co–Re bimetallic particles and pure Re-species with some Co-species also present.

After pre-treatment in H_2_/Ar the major contribution is Co and Re phases, with only a very small presence of Co–Re bimetallic phase. The higher refined sum of first shell multiplicities for CoRe_1.6_ after pre-treatment in H_2_/Ar also indicates a larger particle size for this catalyst. This is in accordance with previous findings on supported Co/Re systems where the role of rhenium is to prevent cobalt agglomeration via promotion of reduction at lower temperatures [10]. Clearly, the latter is true for the CoRe_1.6_ system for pre-treatments in both H_2_/N_2_ and H_2_/Ar, as cobalt undergoes complete reduction starting at a temperature as low as 250 °C. However, the choice of pre-treatment atmosphere clearly affects the Co–Re alloying process, which is significantly inhibited in H_2_/Ar.

### Can In Situ-XAS Reveal Mechanistic Aspects of Cobalt Rhenium During Ammonia Synthesis?

It is assumed that the activity of cobalt rhenium for ammonia synthesis is governed by activation of N_2_ by the active phase, and that the activity of the sample pre-treated in H_2_/N_2_ is superior to that pre-treated in H_2_/Ar, even after the lag. From the in situ EXAFS analysis it is clear that the sample pre-treated in H_2_/N_2_ has better mixture of the Co–Re pair, or consists of more of the bimetallic phase. Significant nitride formation as a result of H_2_/N_2_ pre-treatment can also be excluded, as a Re–N/Co–N shell was not found in EXAFS refinements at any stage. This is further confirmed by XANES and variations in E_0_, which revealed that both Co and Re are completely reduced at all stages. This also correlates with the denitridation of the N_2_/H_2_ pre-treated CoRe_1.6_ sample measuring the ammonia production in H_2_/Ar at 400 °C (Figure S.3). Assuming nitrogen is stored in the material one would expect ammonia production to occur for some time, however this is not observed suggesting a surface reaction mechanism. It should be noted that during fitting procedures, attempts were made to add both a Re–N and Co–N shell around 1.75–1.80 Å to the CoRe_1.6_ at each stage of reaction. For the sample treated in H_2_/N_2_ addition of such a shell at the rhenium edge lowered the R-factor by up to 10%, but very low multiplicities and/or negative Debye–Waller factors were obtained. Such a reduction of the R-factor was also observed for CoRe_1.6_ for both pre-treatments after 60 min in reaction gas. It is possible that a signal in this lower R-range stems from nitrogen inclusion species (M–N_in_) in the metal phase or traces of surface nitrogen species (M–N_surf_), however we believe the average coordination number could be limited by the particle size of the CoRe_1.6_ phase.

## Conclusions

A CoRe_1.6_ catalyst active for ammonia production at 400 °C and ambient pressure following a reduction step at 600 °C in either H_2_/N_2_ or H_2_/Ar was investigated in situ using XAS and XRD. Specifically the reduction profiles were targeted depending on pre-treatment mixture and the nature of the active species during ammonia synthesis reaction conditions analysed. While both pre-treatments render the material active, the H_2_/Ar reduction step results in a 20 min lag-time also observed during in situ measurements. Rhenium XANES reveal a synergistic effect and similar reduction profiles for both pre-treatments, with Co initiating reduction and Re being near completely reduced within the 300–375 °C interval. The only observable difference is in the cobalt XANES, where slight differences are observed between the two pre-treatments as the profile of the H_2_/Ar pre-treatments initiates at a lower temperature. The two pre-treatments result in the formation of Co–Re bimetallic alloys and pure Co and Re monometallic phases. The bimetallic Co–Re phase is the major component following pre-reduction in H_2_/N_2_ while the latter is the major component in the active material following H_2_/Ar pre-treatment. Clearly the presence of nitrogen in the reduction step promotes a high degree of bimetallic Co–Re mixing confirmed by Co–Re/Re–Co absorption shells at both edges. EXAFS confirms restructuring of the pure Re and Co phases following H_2_/Ar pre-treatment only after 60 min during time on stream in H_2_/N_2_. Formation of the bimetallic Co–Re phase coincides with the observed lag-time both during catalytic experiments and in situ conditions.

## Electronic Supplementary material

Below is the link to the electronic supplementary material.

Supplementary material 1 (DOCX 735 KB)
