# Supplementary material for: An In Situ XAS Study of the Cobalt Rhenium Catalyst for Ammonia Synthesis
Source: Top Catal. 2018 Feb 12;61(3):225–39. doi: 10.1007/s11244-018-0892-7 (PMC6413814; doi:10.1007/s11244-018-0892-7)
Supplement: Supplementary file 1 — Supplementary material 1 (DOCX 735 KB) [file 11244_2018_892_MOESM1_ESM.docx]

## An *in situ* XAS study of the cobalt rhenium catalyst for ammonia synthesis

Karina Mathisen^1^, Karsten Kirste^1^, Justin S. J. Hargreaves^2^, Said Laassiri^2^, Kate McAulay^2^, Andrew R. McFarlane^2^ and Nicholas A. Spencer^2^.

^1^Department of Chemistry, Norwegian University of Science and Technology, Høgskoleringen 5, N-7491 Trondheim, Norway

^2^WestCHEM, School of Chemistry, Joseph Black Building, University of Glasgow, Glasgow G12 8QQ, UK

### Supplementary information


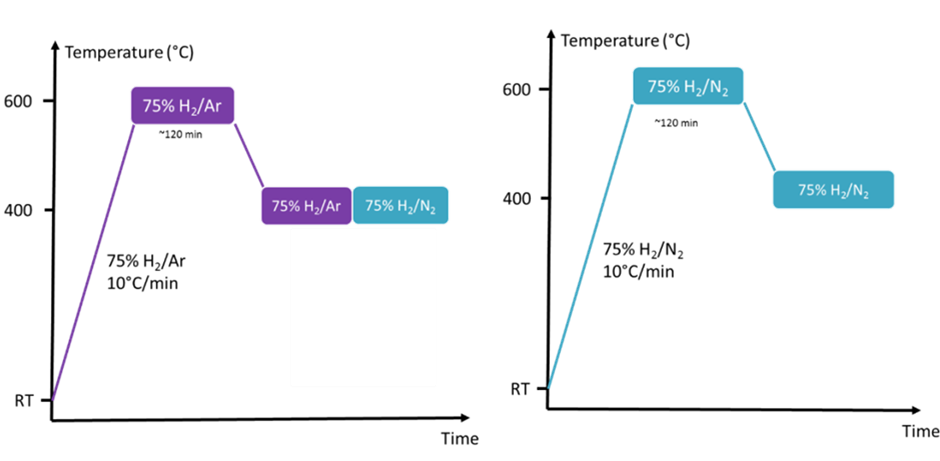


**Figure S.1** The two protocols used for the *in situ* XAS/XRD study of cobalt rhenium for ammonia synthesis


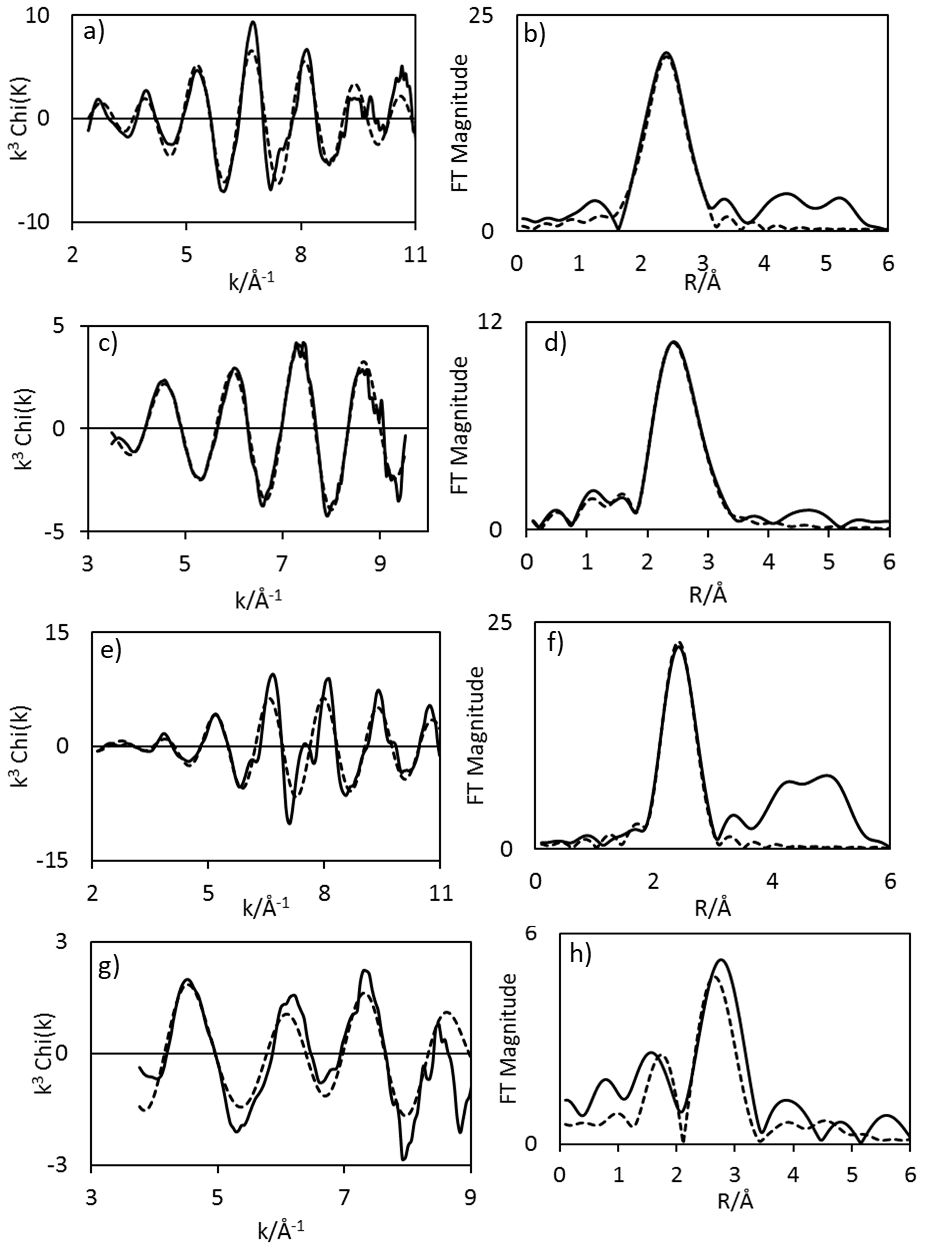


**Figure S.2** Experimental (−) and calculated (---) k^3^-weighted EXAFS (left) and its Fourier Transform (right) for reference compounds: CoRe_y_N_x_ (a-b) Re L_III_-edge and (c-d) Co K-edge, CoN_x_ (e-f) for the Co K-edge and ReN_x_ (g-h) Re L_III_-edge.


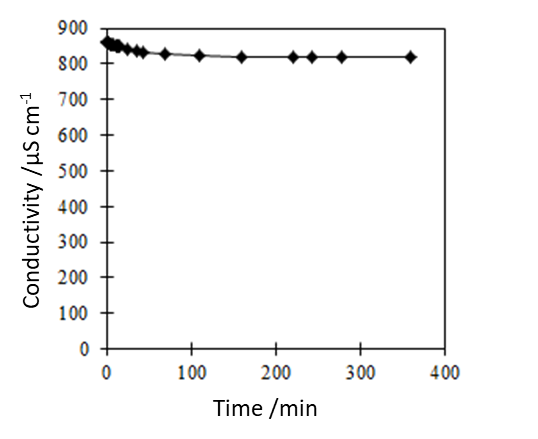


**Figure S.3** Ammonia yield of CoRe_1.6_ using 75% H_2_ in Ar (BOC, 99.98%) at a total gas feed of 60 ml min^-1^ at 400°C


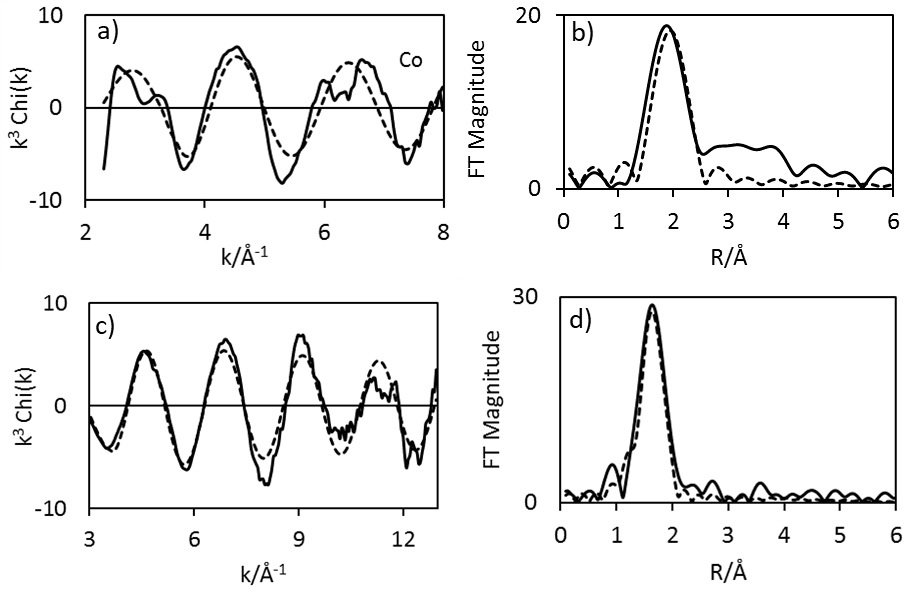


**Figure S.4** Experimental (−) and calculated (---) k^3^-weighted EXAFS (left) and its Fourier Transform (right) for the fresh CoRe_1.6_ at the Co K-edge (a-b) and the Re L_III_-edge (c-d).


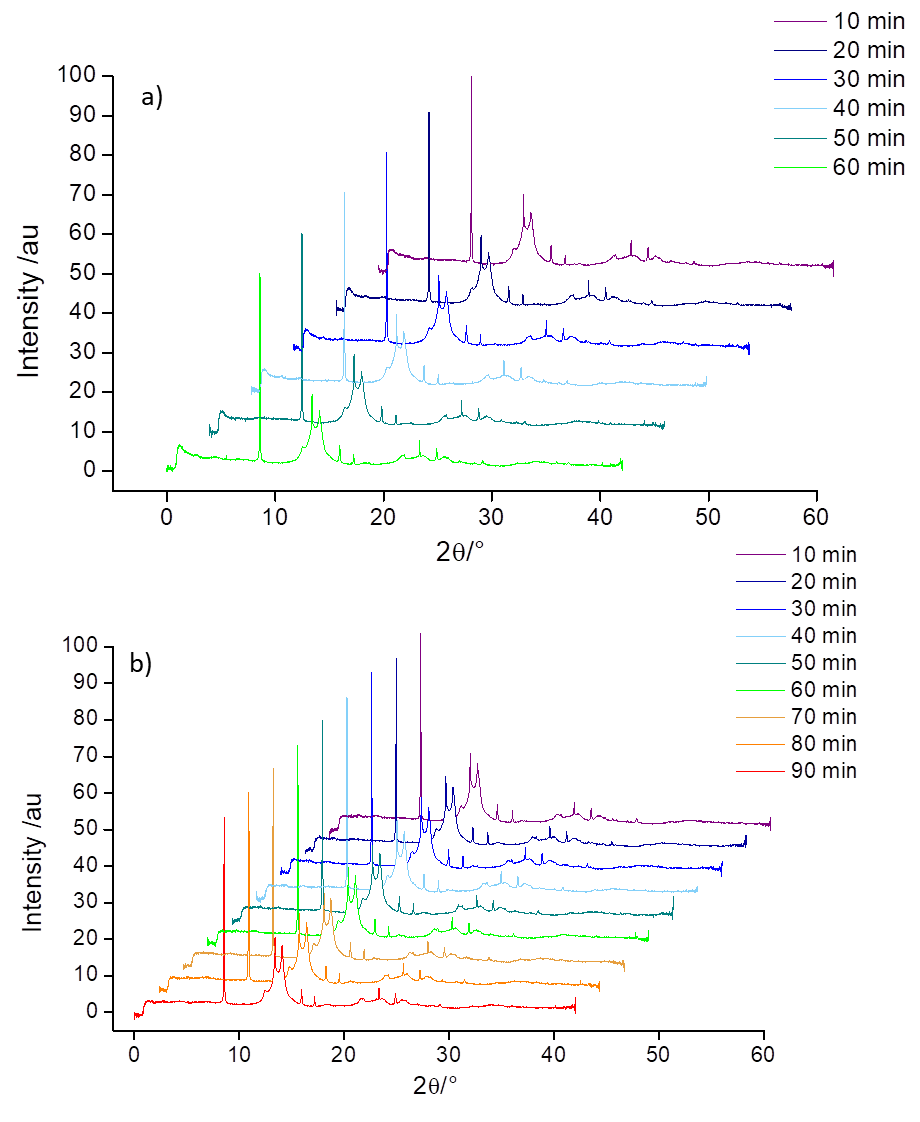


**Figure S.5** *In situ* XRD a) H_2_/N_2_ pre-treatment and b) H_2_/Ar pre-treatment.


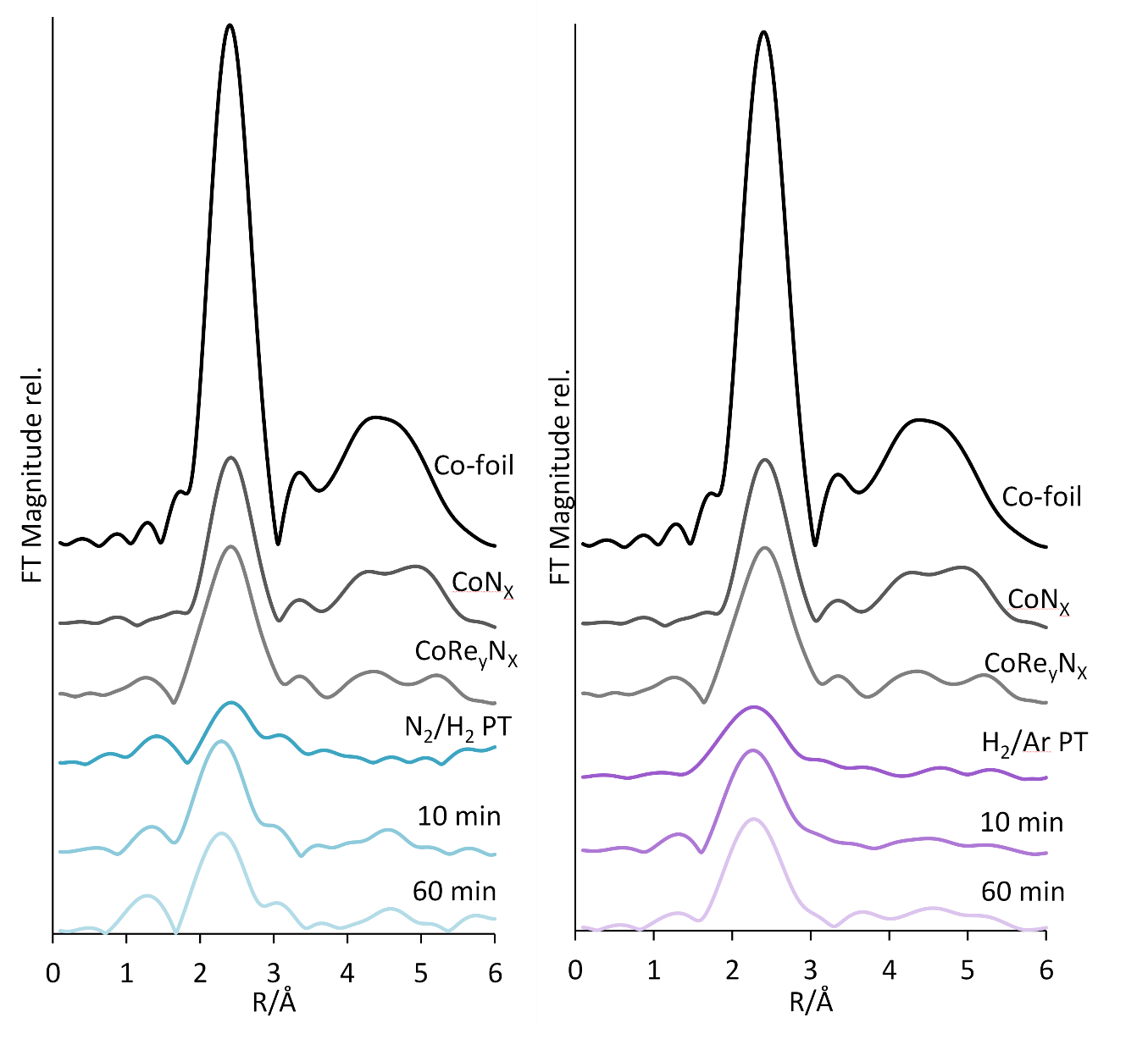


**Figure S.6** Experimental k^3^-weighted Fourier Transform for CoRe_1.6_ after pre-treatment in H_2_/N_2_ (left) and H_2_/Ar (right) and after two ammonia synthesis reaction times at 400°C for the Co K-edge compared to references.


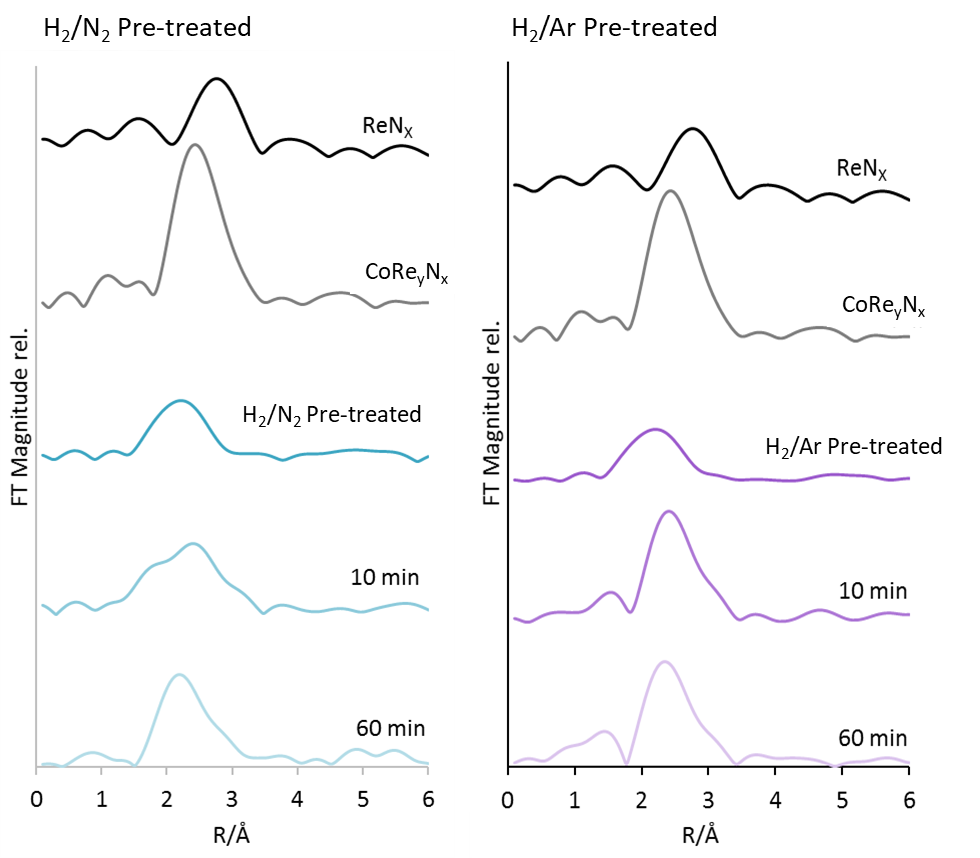


**Figure S.7** Experimental k^3^-weighted Fourier Transform for CoRe_1.6_ after pre-treatment in H_2_/N_2_ (left) and H_2_/Ar (right) and after two ammonia synthesis reaction times at 400°C for the Re L_III_-edge compared to references.
